# Retinal thickness and vascular density changes in Keratoconus: A systematic review and meta-analysis

**DOI:** 10.1016/j.heliyon.2025.e42099

**Published:** 2025-01-18

**Authors:** Hadi Vahedi, Mirsaeed Abdollahi, Reza Moshfeghinia, Shima Emami, Navid Sobhi, Rana Sorkhabi, Ali Jafarizadeh

**Affiliations:** a Nikookari Eye Center, Tabriz University of Medical Sciences, Tabriz, Iran; b Student Research Committee, Shiraz University of Medical Sciences, Shiraz, Iran; c Research Center for Evidence-Based Medicine, Iranian EBM Centre: A Joanna Briggs Institute Affiliated Group, Tabriz University of Medical Sciences, Tabriz, Iran; d Department of Ophthalmology, Faculty of Medicine, Tabriz University of Medical Sciences, Tabriz, Iran

**Keywords:** Keratoconus, Retinal thickness, Vascular density, Optical coherence tomography, Meta-analysis, Systematic review

## Abstract

**Purpose:**

This systematic review and meta-analysis aimed to assess the changes in retinal layer thickness and vascular density in patients diagnosed with keratoconus (KC).

**Methods:**

We conducted a thorough search in PubMed, Scopus, Embase, and Web of Science, encompassing all observational studies from January 1990 to February 2024. We analyzed the data using STATA software 17 and the random effects model with the restricted maximum likelihood (REML) or maximum likelihood (ML) method to pool the standardized mean difference (SMD) and the weighted mean difference (WMD) of retinal thickness and vascular density compared to non-KC eyes.

**Results:**

From 4608 records, 16 primary studies met our inclusion criteria, involving 1932 eyes in two groups. The meta-analysis revealed a significant reduction in retinal nerve fiber layer (RNFL) thickness (SMD: −0.30 [-0.52, −0.08]), perifoveal macular thickness in the nasal region (SMD: −0.32 [-0.55, −0.09]) and superior region (SMD: −0.24 [-0.47, −0.02]), and superior capillary plexus (SCP) density (SMD: −1.52 [-2.27, −0.77]) of KC eyes. Additionally, there was a mildly to moderately significant increase in the optic nerve head (ONH) disk area (SMD: 0.40 [0.06, 0.75]). However, there were no significant changes in foveal macular thickness, parafoveal macular thickness, or deep capillary plexus (DCP) density.

**Conclusion:**

In KC patients, significant alterations in retinal structure and vascular density have been observed. Although these changes do not currently influence treatment strategies, they offer valuable insights into the relationship between the retina and the cornea. Understanding these changes may be essential for future research focused on early detection, potential prognosis markers, and further exploration of the retina-corneal connection in KC.

## Highlights

• Significant RNFL thickness and SCP density reduction are shown in Keratoconus (KC).
• Younger KC patients (<30 years) exhibited more pronounced reduction in RNFL thickness.
• Moderately significant increase in optic nerve head disk area was observed in KC.
• Early detection of retinal changes in KC are important to manage visual impairment.

## 1 Introduction

Keratoconus (KC) is a progressive bilateral asymmetrical disease characterized by corneal thinning resulting in protrusion [1,2]. In this condition, the cornea undergoes a transformation into a conical shape, resulting in a progressive myopic shift and high irregular astigmatism, leading to reduced visual acuity (VA) and progressive visual impairment [3,4].

KC exhibits a global presence, with an estimated prevalence of 138 per 100,000 individuals and an incidence rate of 1.3 per 100,000 [5,6]. While KC can manifest at any age, its prevalence peaks notably in the second decade of life. Notably, there is no gender difference in the incidence or prevalence [5,7]. The total economic burden of treating KC in 2019 was USD 3.8 billion, with an average lifetime cost of USD 28,766.69 [8]. Among the most common reasons for corneal transplantation globally, KC accounts for 31 percent of procedures in Australia and 15 percent in the United States [6]. Due to its prevalence among young people, KC has significant implications for the education and personality development of affected individuals [9].

Extensive previous evidence suggests that KC can be associated with both structural and functional changes in different parts of the posterior segment of the eye, such as the choroid and retina [[10], [11], [12], [13]]. By using different optical coherence tomography (OCT) devices, studies have identified alterations in retinal layer thickness, particularly in the central macular region and paracentral retinal region. Moreover, significant changes in different layers of the sensory retina, including the retinal nerve fiber layer (RNFL), retinal ganglion cell layer (RGCL), retinal pigment epithelium (RPE) layer, and optic nerve head (ONH) parameters, were identified between individuals with KC and those without the condition [6,7,[13], [14], [15]]. Moreover, OCT-angiography (OCTA) examinations have demonstrated alterations in retinal and choroidal vessel density in the KC, perhaps due to vascular remodeling or altered perfusion [10].

Based on our knowledge, no previous study has been conducted to comprehensively assess the changes in retinal layer thickness or vessel densities in KC. In addition, previous primary studies sometimes had various and inconsistent results. Therefore, we aimed to systematically review these changes based on OCT or OCTA results. Finally, we performed a meta-analysis to combine these results.

## 2 Materials and methods

The design and conduct of this study were performed according to the Cochrane Handbook for Systematic Review [16]. The review was reported in accordance with the standards outlined in the Preferred Reporting Items for Systematic Reviews and Meta-Analysis Statement (PRISMA) [17].

### 2.1 Protocol and registration

The protocol of this study was designed based on the predefined approach, which was registered in the International Prospective Register of Systematic Reviews (PROSPERO) database. The registration number is CRD42023476974.

### 2.2 Information source and search Strategy

A systematic search was conducted to find studies on retinal changes in KC using OCT/OCTA. After determining the search keywords, we searched both MeSH and free text keywords in PubMed, Scopus, Embase, and Web of Science. As grey literature, we searched Scopus and ProQuest to identify additional documents. All searches were performed manually from January 1990 (the emergence of OCT/OCTA) to February 2024. No restrictions based on language or geographical area were applied. The full search syntax for different databases and excluded articles from the full-text screening stage are shown in Supplementary Tables S1 and S2.

### 2.3 Eligibility criteria

The study employed the Population, Exposure, Comparison, Outcome, and Study Design (PECOS) criteria to establish the inclusion and exclusion criteria, as well as the research questions. All published human observational articles from January 1990 to February 2024 encompassed cross-sectional, prospective or retrospective cohort, and case-control studies (Study design), in a population with confirmed KC that includes all ages, races, and genders (Population), assessing the association between KC (Exposure), and retinal thickness and vascular density changes (Outcome), compared to eyes without KC (Comparison). The exclusion criteria were republished articles with the same sample, books, literature reviews, newspapers, notes, surveys, letters, editorials, clinical trials, case reports, conference abstracts, unpublished manuscripts, and animal studies.

### 2.4 Selection process

The titles and abstracts of all studies were screened by S.E. in order to identify any studies that could be relevant to this systematic review. Afterward, the complete texts of the studies were evaluated independently by two researchers (N.S.) and (H.V.) to determine which met the inclusion and exclusion criteria and were therefore eligible for inclusion. Disagreements were resolved through discussion; in cases where different opinions remained unresolved, the third expert researcher (M.A.) made the final decision.

### 2.5 Quality assessment (risk of bias assessment)

The risk of bias (ROB) was assessed by the two authors (N.S.) and (H.V.) independently, using the Newcastle-Ottawa Scale (NOS) adapted for cross-sectional studies [18]. Disagreements between them were resolved through discussion or by the third author (M.A.). The NOS includes three domains, including selection (points from 0 to 5), comparability (0–2), and outcome (0–3). Then, studies were categorized according to the total scores: very high (0–3 points), high (4–6 points), and low risk of bias (7–10 points).

### 2.6 Data extraction

An extraction template was formulated according to the research aims and subsequently finalized after testing on at least one study. The data from each primary study was then separately collected by S.E. and H.V., and any discrepancies were resolved by consensus. The information related to the selected studies, including the author's name, year of publication, location, study sample size, demographic data of subjects, OCT/OCTA device models, and their results, was recorded in the designed form. Regarding the staging of KC, all included studies either applied the Amsler-Krumeich classification directly or reported keratometry measurements consistent with this classification, ensuring a consistent approach to staging across studies, despite geographic differences.

### 2.7 Statistical analysis

The measured outcomes were pooled zonal vessel densities in the macula or peripapillary area as a percentage, foveal avascular zone (FAZ) area, rim, disk, and cup area in mm^2^, cup volume of optic nerve head (ONH), and cube volume of the macula in mm^3^, retinal zonal thickness in the macula, RNFL thickness, and retinal ganglion cell layer (RGCL) thicknesses in μm, if information was provided.

Meta-analyses were performed using the ‘meta’ package in STATA software version 17 (StataCorp, College Station, Texas, USA). The random effects model (REM), which is more conservative to compensate for methodological differences between studies, with the restricted maximum likelihood (REML) or maximum likelihood (ML) method, was applied to estimate the pooled standardized mean difference (SMD) and the weighted mean difference (WMD) as the effect sizes (ES) with a 95 % confidence interval (CI). The WMD was used to measure the absolute difference between the mean values in two groups, while the SMD was used to compare the assessments and was reported as Hedges’ g to correct for possible overestimation of small sample bias and was categorized as trivial (≤0.19), mild (0.2–0.49), moderate (0.5–0.79), and high (≥0.80) [19]. In cases where primary studies lacked complete data or had missing values for the standard deviation (SD), we imputed the SD by referencing the nearest comparable study, taking into account the respective sample sizes [20].

Statistical heterogeneity among studies was assessed using the I^2^ statistic and Q test (χ2) with a significance level of p-value≤0.050 [21]. The interpretation of I^2^ values was delineated as follows: 0%–40 % indicating that heterogeneity might not be significant; 30%–60 % suggesting the presence of moderate heterogeneity; 50%–90 % indicating the likelihood of substantial heterogeneity; and 75%–100 % suggesting considerable heterogeneity [16].

Subgroup analysis was carried out to determine the possible source of the heterogeneity across studies based on age, risk of bias, and area (continent). In addition, sensitivity analysis was performed using the leave-one-out method to evaluate the robustness of the pooled estimates.

The potential presence of publication bias or small study effect was assessed through contour-enhanced funnel plots (if 10 studies or more were identified for the measured outcome) and utilizing Egger's regression test. Finally, the trim-and-fill method was used to correct the possible publication bias if there was a significant finding (p-value<0.1).

### 2.8 Certainty of the evidence

To determine the certainty of evidence in our study, we utilized the GRADE (Grading of Recommendations, Assessment, Development, and Evaluation) methodology [22]. This system classifies evidence into four categories: high, moderate, low, and very low. Evidence can be downgraded based on five domains: risk of bias, inconsistency, indirectness, imprecision, and publication bias. Conversely, evidence can be upgraded based on three criteria: large magnitude of association, dose-response gradient, and residual plausible bias and confounding. We assessed indirectness by examining whether the evidence directly addressed the relevant population, intervention, comparison, and outcomes. Imprecision was evaluated by analyzing confidence intervals, where wider intervals indicated less precision. Additionally, we investigated publication bias to determine if selective publication might influence the results. The final evidence quality was categorized as high, moderate, low, or very low, reflecting our confidence in the effect estimate and its proximity to the true effect. This systematic approach ensured that our recommendations were clear and reliable, grounded in the strength and quality of the available evidence.

## 3 Results

### 3.1 Study selection

**Fig. 1:**
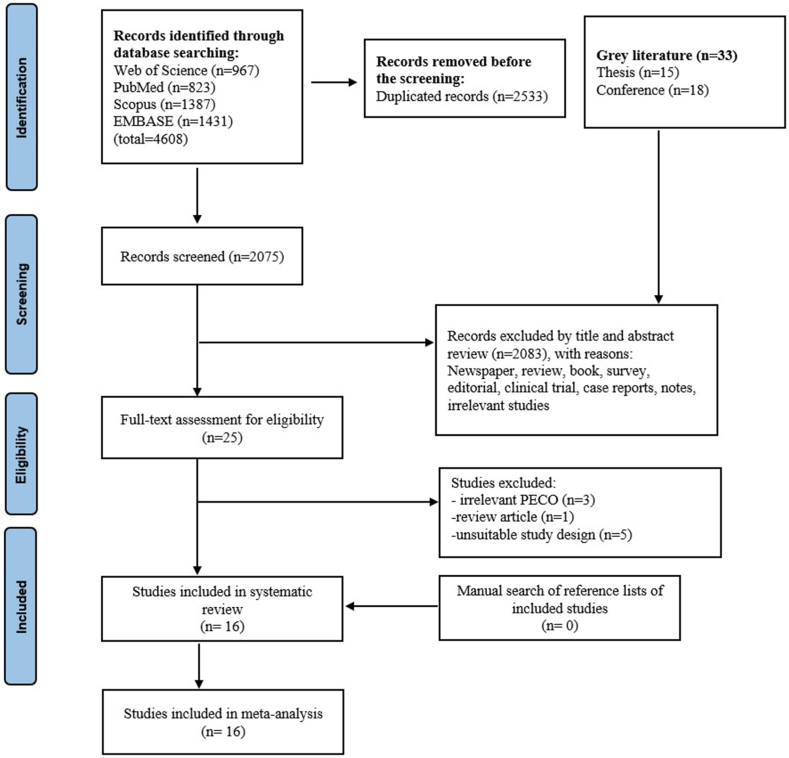
The Preferred Reporting Items for Systematic Reviews and Meta-Analyses (PRISMA) flow diagram.

The PRISMA flowchart of this study is presented in Fig. 1. A total of 4608 records were identified after conducting a search in all official databases. Prior to screening, 2533 studies were removed due to duplications, and 2075 unique studies were identified. Twenty-five studies were selected for full-text assessment. Finally, 16 studies met our inclusion criteria and were included in this study [1,2,7,14,15,[23], [24], [25], [26], [27], [28], [29], [30], [31], [32], [33]]. After forward and backward citation tracking of these studies, no more articles were identified.

### 3.2 Study Characteristics

Table 1 provides a summary of the main features of the 16 studies that were included. All included studies were designed as cross-sectional studies, which were published between 2012 and 2023 and encompassed 1932 eyes in two groups (eyes with and without KC). The sample size of the included studies ranged from 56 to 303. Eight studies were done in Asia [2,7,14,15,23,24,28,31], mostly in Turkey, four in Europe [1,[25], [26], [27]], two in Africa [29,32], one in Australia [33], and one in the United States [30].

**Table 1:** Main features of included studies.

Article (year)	Mean Age (SD)	Male Ratio	Number of Eyes	OCT/OCTA device	Population exclusion criteria	OCT-A exclusion criteria
Dogan et al., 2023 [23]	KC: 28.50 (11.48) Control: 30.63 (8.17)	N/A	KC: 32 Control: 24	Optovue RTVue XR 100 Avanti, Fremont	1. Previous history of any ocular surgery and any ocular or systemic disease. 2 Patients using rigid gas-permeable contact lenses. 3. Patients using soft contact lenses were asked to discontinue use for 2 weeks before the examination.	1. Only images with a high signal strength (≥7/10) were accepted. 2 Patients with poor image features, poor clarity, blink artifacts, poor fixation causing residual motion or doubling artifacts, and segmentation errors, were excluded from the analysis.
Zırtıloglu et al., 2023 [31]	KC: 30.0 (8.0) Control: 31.1 (7.5)	KC: 61.2 % Control: 58.1 %	KC: 67 Control: 74	AngioVue software RTVue-XR, Avanti version 2018.0.0.14; Optovue, Fremont	1. Non-keratoconus Ocular Diseases 2 Optic Nerve Disease 3 Ocular Trauma and Surgery 4 Eyes classified as Stage 4 were excluded due to low image quality (signal strength index <8/10). 5. If both eyes were eligible, one eye was chosen at random for each subject.	N/A
Wylegała 2022 [1]	KC: 43.64 (15.30) Control: 40.10 (14.28)	55.71 %	KC: 79 Control: 47	Zeiss Angioplex software	1. Patients using contact lenses were told to abstain from wearing them for 72 h before the examination. 2. Only Polish Caucasians without systemic diseases were included in the analysis. 3. Patients with other anterior segment disorders except KC and posterior segment diseases.	N/A
Pierro et al, l 2022 [25]	KC: 26.92 (9.6) Control: 26.66 (1.8)	KC: 67 % Control: 67 %	KC: 32 Control: 32	SS DRI Triton (Topcon, Tokyo, Japan)	1. Patients were told not to use contact lenses at least 72 h before the examination. 2. Spherical equivalent outside the range of ± 8 diopters. 3. History of ocular disease or surgery, systemic diseases potentially able to affect corneal and retinal tissues 4. Eyes in which a full set of high-quality acquisitions could not be acquired properly.	1. If the lamina cribrosa was not visible on both horizontal and vertical scans by two masked graders (AS, AD) or the OCTA acquisition was largely affected by motion or blinking artifacts that could potentially interfere with image processing, the eye was excluded from further analysis. 2. Fifty-four eyes (63 %) had poor-quality images and were excluded from the final analysis.
Cankaya et al., 2012 [14]	KC: 29.9 (6.8) Control: 31.5 (6.8)	KC: 43.5 % Control: 31.1 %	KC: 68 Control: 85	Spectralis (Heidelberg Engineering)	1. Evidence of secondary causes of elevated IOP, presence of any retinal disease, neurologic diseases affecting the optic disc or visual ﬁeld, and history of any surgery or laser procedures 2. Patients with tilted discs, non-glaucomatous optic disc atrophy, or any signiﬁcant media opacity in which the fundus was not visible	N/A
Deonarain et al., 2019 [32]	KC: mild: 25.53 (4.24) moderate: 25.45 (6.30) severe: 23.33(3.56) Control: 24.61 (4.41)	KC: mild: 80 % moderate: 45.45 % severe: 61.11 % Control: 63.63 %	KC: mild: 15 moderate: 11 severe: 18 Control: 44	Optovue iVue100	1. Patients presenting with ocular pathology other than keratoconus, systemic conditions 2. Patients who have a history of ocular surgery and/or injury, have intraocular pressure higher than 21 mmHg and have central corneal scarring	N/A
Yilmaz et al., 2017 [24]	KC: 12.4 (1.9) Control: 12.0 (2.1)	KC: 56 % Control: 52 %	KC: 50 Control: 50	Spectralis (Heidelberg Engineering)	1. History of any ocular disease other than keratoconus and any systemic disease 2. History of previous intraocular surgery including corneal cross-linking or laser therapy 3. Presence of any corneal scarring/opacity which may influence OCT imaging 4. Using contact lenses within the last month. 5. > 15 years old	N/A
Cuppusamy et al., 2018 [29]	KC: 24.29 (5.02) Control: 24.11 (4.67)	KC: 50 % Control: 50 %	KC: Rt: 19 Lt: 24 Control: Rt: 19 Lt: 24	Optovue iVue100	1. Patients with systemic and/or ocular diseases other than keratoconus 2. History of ocular surgery and/or trauma 3. IOP >21 mmHg 4. Central corneal scarring and advanced keratoconus (K1 and/or K2 greater than 54 diopters with corneal scarring)	N/A
Sahebjada et al., 2015 [33]	KC: 35 (13.5) Control: 44.25 (15.0)	KC: 62 % Control: 38.5 %	KC: 129 Control: 174	Carl Zeiss Meditec, model 3000; software version 4.0	Potential subjects with non-keratoconus ocular disease in both eyes	1. Scans were accepted if free of artifacts (boundary errors and decentration), and complete cross-sectional images were seen for all individual line scans. 2. Only scans with signal strengths of at least 5 out of 10 were used
Brautaset et al., 2015 [30]	KC: 37.3 (12.2) Control: 37.6 (12.6)	KC: 50 % Control: 48.07 %	KC: 48 Control: 52	Zeiss Cirrus HD-OCT	Any previous ocular surgery, the use of any systemic or ocular medications, and any chronic disorder that can affect the eye	N/A
Reibaldi 2012 [27]	KC: Stage I: 27 [8] Stage II: 28 [6] Stage III: 30 [7] Control: 29 [4]	KC: Stage I: 72.72 % Stage II: 71.05 % Stage III: 63.63 % Control: 44.44 %	KC: Stage I: 26 Stage II: 38 Stage III: 11 Control: 36	Spectralis software version 4.0	1. Patients using contact lenses for the correction of refractive error were instructed to discontinue their use before the examination, for at least 2 weeks for soft contact lenses and at least 4 weeks for rigid gas-permeable contact lenses. 2. Patients with other ocular pathologies affecting the cornea, previous uveitis, ocular surgery or trauma, retinal or macular pathology, tilted discs, peripapillary atrophy, and any neurological disease were excluded from the study	1. Scans with a quality of less than 15 (as suggested by the manufacturer) were repeated until good quality was achieved. If the quality of the scans was less after three attempts, the participant was excluded from the analysis. 2. Likewise, scans with blinks during the scanning process were excluded and repeated.
Orman et al., 2021(2)	KC: 25.5 (1.36) Control: 26.7 (1.13)	KC: 59.61 % Control: 50 %	KC: 52 Control: 50	SD-OCT Scan (Nidek Inc., CA, USA)	A signal strength index >7 was included	N/A
Hashemian et al., 2023 [28]	KC: 25.6 (7.4) Control: 31.7 [6]	KC: 58.8 % Control: 33.3 %	KC: 68 Control: 72	Spectralis (Heidelberg Engineering)	1. Any previous ocular surgery, corneal diseases other than KC, uveitis, uncontrolled glaucoma, severe media opacity, visual acuity of <20/200, and systemic or topical use of vasoactive medications which may affect the choroidal blood circulation. 2. The control group was composed of healthy individuals who qualified for refractive surgery as they had myopia <6 diopters and otherwise normal eyes with normal corneal imaging.	Poor-quality scans, in which the sclerochoroidal junction was not discernible
Moschos et al., 2013 [26]	KC: 33.9 (8.7) Control: 34.4 (7.2)	KC: 59.37 % Control: 56.66 %	KC: 64 Control: 60	Carl Zeiss, model 3000, Meditec, Retinal mapping software	Any disorders that might affect the OCT and mf-ERG recording: cataract and optical media opacities, glaucoma, generalized or inherited retinal dystrophies, retinal detachment, degenerative myopia, high refractive error, microphthalmos	N/A
Uzunel et al., 2016 [15]	KC: Grade 1: 29.7 (8.1) Grade 2: 31.6 (9.4) Grade 3: 33.8 (10.7) Control: 29.1 (7.9)	KC: 39.7 % Control: 48.3 %	KC: Grade 1: 29 Grade 2: 29 Grade 3: 26 Control: 29	Cirrus HD OCT, with software version 6.0.2.81	1. The fellow eyes of 52 patients were excluded from the study for the following reasons: corneal scars (12 eyes), previous penetrating keratoplasty (10 eyes), cross-linking (18 eyes), and no discontinuation of contact lens use one week before the measurement (12 eyes). 2. Patients with Grade 4 KC were not included in the study because of corneal scarring.	N/A
Özsaygıl et al., 2021 [7]	KC: Stage 1: 26.25 (7.14) Stage 2: 27.32 (7.43) Stage 3: 29.94 (10.34) Control: 26.25 (7.14)	KC: 48.14 % Control: 47.5 %	KC: Stage 1: 40 Stage 2: 27 Stage 3: 18 Control: 40	Spectralis (Heidelberg Engineering)	1. Presence of non-KC corneal or retinal or optic nerve pathology in either eye and any systematic disease 2. Age less than 18 or over 40 years 3. Myopia greater than −6 diopters 4. Axial length longer than 26 mm	1. Patients with severe corneal problems that precluded SD-OCT or Pentacam imaging and participants with vitreoretinal interface pathology (e.g., vitreoretinal traction, retinoschisis, epiretinal membrane, and lamellar macular hole) that could affect segmentation in SD-OCT scans were excluded from the study 2. Patients who had undergone corneal crosslinking were also excluded due to its potential effects on the retinal layers.

Studies encompassed demographically diverse individuals of both genders, with average ages of ≥12 years old. However, there was a wide variability in the age distribution among the study participants. The diagnostic modalities employed across all investigations consisted of slit lamp examinations or topography assessments. The inclusion criteria for each study were similar, and generally, they excluded patients with a previous history of any eye disease or using contact lenses as potential confounding factors.

### 3.3 Risk of bias assessment

According to the NOS, the articles included in the analysis had a total quality score ranging from 4 to 8. Nine studies were classified as having a “Low” risk of bias, while seven studies were classified as having a “High” risk of bias. No study was found to have a “Very High” risk of bias. All research employed a valid method for KC determination, although none of the studies employed a clear random sampling technique for participant selection, and all studies were conducted in Hospital settings. Moreover, all these articles lacked clarity regarding the response rate. Table 2 contains the responses to questions in each field for all the studies that were included.

**Table 2:** New-castle-Ottawa score for cross-sectional studies.

Article (year)	Selection				Comparability	Outcome		Total Score
	Representativeness of the cases	Sample size	Ascertainment of the screening/surveillance tool	Non-response rate	The potential confounders were investigated by subgroup analysis or multivariable analysis	Assessment of the outcome	Statistical test	
Wylęgała et al., 2009 [1]	–	∗	–	–	∗	∗∗	∗	5
Uzunel et al., 2016 [15]	–	∗	–	–	∗	∗∗	∗	5
Dogan et al., 2022 [23]	–	∗	∗∗	–	–	∗∗	∗	6
Cankaya et al., 2012 [14]	–	∗	∗∗	–	–	∗∗	∗	6
Özsaygılı et al., 2020 [7]	–	∗	∗∗	–	–	∗∗	∗	6
Orman et al., 2021 [2]	–	∗	∗∗	–	∗	∗∗	∗	7
Yilmaz et al., 2017 [24]	–	∗	∗∗	–	∗	∗∗	∗	7
Pierro et al., 2022 [25]	–	∗	–	–	–	∗∗	∗	4
Reibaldi et al., 2012 [27]	–	∗	∗∗	–	–	∗∗	∗	6
Hashemian et al., 2023 [28]	–	∗	∗∗	–	∗	∗∗	∗	7
Brautaset et al., 2015 [30]	–	∗	∗∗	–	∗	∗∗	∗	7
Moschos et al., 2013 [26]	–	∗	∗∗	–	∗∗	∗∗	∗	8
Cuppusamy et al., 2018 [29]	–	∗	∗∗	–	∗	∗∗	∗	7
Deonarain et al., 2019 [32]	–	∗	∗∗	–	∗	∗∗	∗	7
Zırtılog lu et al., 2022 [31]	–	∗	∗∗	–	∗	∗∗	∗	7
Sahebjada et al., 2015 [33]	–	∗	∗∗	–	∗∗	∗∗	∗	8

### 3.4 Data synthesis

The results of the meta-analysis of the included studies on defined outcomes are summarized in Table 3 (Detailed plots of meta-analyses are shown in Supplementary Figs. S1–S29.).

**Table 3:** Pooled effect sizes and 95 % confidence intervals (CI) for measured outcomes^a^.

Outcomes	Overall Effects						No. of studies	No. of eyes	Egger's
	Hedges' g with 95%CI	P-value (Q)	I^2^ (%)	WMD with 95%CI	P-value (Q)	I^2^ (%)			
Macular whole thickness	−0.34 [-0.64, −0.04]	0.14	8.38	−6.44 [-16.27, 3.38]	0.05	72.95	2	213	–
Foveal macular thickness	0.43 [-0.23, 1.08]	0.00	97.1	3.28 [-0.67, 7.22]	0.00	80.17	12	1537	0.97
Parafoveal macular thickness (overall)	1.79 [-2.10, 5.68]	0.00	99.56	1.99 [-7.10, 11.08]	0.00	94.52	3	500	0.55
Parafoveal macular thickness (inferior)	−0.07 [-0.30, 0.15]	0.47	0.00	−1.49 [-5.37, 2.38]	0.51	0.00	3	290	0.85
Parafoveal macular thickness (nasal)	−0.10 [-0.33, 0.13]	0.52	0.00	−1.76 [-6.21, 2.69]	0.49	0.00	3	290	0.43
Parafoveal macular thickness (superior)	−0.12 [-0.34, 0.11]	0.40	0.00	−3.06 [-7.48, 1.37]	0.61	0.00	3	290	0.88
Parafoveal macular thickness (temporal)	−0.05 [-0.27, 0.18]	0.53	0.00	−0.94 [-5.09, 3.21]	0.54	0.00	3	290	0.74
Perifoveal macular thickness (overall)	2.96 [-1.04, 6.96]	0.00	99.16	5.99 [0.72, 11.27]	0.00	73.2	2	359	–
Perifoveal macular thickness (inferior)	−0.09 [-0.32, 0.14]	0.64	0.00	−1.32 [-5.42, 2.78]	0.58	0.00	3	290	0.49
Perifoveal macular thickness (nasal)	−0.32 [-0.55, −0.09]	0.69	0.00	−5.94 [-10.69, −1.19]	0.31	0.00	3	290	0.92
Perifoveal macular thickness (superior)	−0.24 [-0.47, −0.01]	0.64	0.00	−3.80 [-7.32, −0.29]	0.66	0.00	3	290	0.40
Perifoveal macular thickness (temporal)	−0.12 [-0.38, 0.14]	0.15	21.99	−1.97 [-7.21, 3.27]	0.15	47.17	3	290	0.18
RNFL (overall)	−0.30 [-0.52, −0.08]	0.01	63.12	−3.15 [-5.73, −0.56]	0.00	81.66	9	975	0.19
RNFL stage 1	−0.35 [-0.63, −0.07]	0.82	0.00	−1.39 [-3.08, 0.29]	0.29	25.61	3	200	0.79
RNFL stage 2	−0.24 [-1.06, 0.58]	0.00	87.93	−3.42 [-12.04, 5.20]	0.00	94.89	3	199	0.02
RNFL stage 3	−0.72 [-1.88, 0.44]	0.00	90.70	−8.14 [-22.30, 6.03]	0.00	96.78	3	160	0.72
GCL (overall)	−0.56 [-1.40, 0.27]	0.00	88.13	−4.94 [-12.72, 2.84]	0.00	90.93	2	238	–
GCL stage 1	−0.47 [-1.02, 0.07]	0.11	61.08	−3.10 [-5.57, −0.62]	0.25	25.69	2	138	–
GCL stage 2	−0.47 [-1.35, 0.41]	0.01	83.35	−3.78 [-10.81, 3.25]	0.01	84.47	2	125	–
GCL stage 3	−0.83 [-2.2, 0.54]	0.00	91.26	−8.57 [-23.36, 6.23]	0.00	94.13	2	113	–
ONH cup area	0.36 [0.11, 0.61]	0.79	0.00	0.17 [0.05, 0.28]	0.88	0.00	2	255	–
ONH disk area	0.40 [0.06, 0.75]	0.03	67.10	0.19 [0.08, 0.30]	0.17	40.71	4	437	0.71
ONH rim area	0.28 [-0.10, 0.66]	0.06	63.99	0.10 [-0.02, 0.21]	0.09	57.10	3	335	0.78
ONH cup volume	0.24 [0.01, 0.48]	0.40	0.00	0.06 [-0.04, 0.16]	0.11	60.72	2	279	–
Macular cube volume	−0.11 [-0.46, 0.24]	0.02	72.04	−0.12 [-0.36, 0.12]	0.01	75.87	4	480	0.00
Macular whole vessel density SCP	−1.52[-2.27, −0.77]	0.00	85.03	−4.81 [-6.79, −2.83]	0.01	82.44	3	261	0.91
Macular whole vessel density DCP	−0.96 [-2.57, 0.65]	0.00	96.86	−5.09 [-12.39, 2.22]	0.00	98.12	3	261	0.71
ONH whole vessel density	−0.52 [-0.80, −0.23]	0.95	0.00	−1.25 [-1.92, −0.58]	0.91	0.00	2	197	–
ONH peripapillary vessel density	0.14 [-0.40, 0.67]	0.01	76.97	0.51 [-1.26, 2.28]	0.01	80.81	3	261	0.47
FAZ area	−0.27 [-0.70, 0.17]	0.16	48.60	−0.03 [-0.08, 0.02]	0.11	59.82	2	182	–

a Detailed plots of meta-analyses are shown in Supplementary Figs. S1–S29.

#### 3.4.1 RNFL thickness

**Fig. 2:**
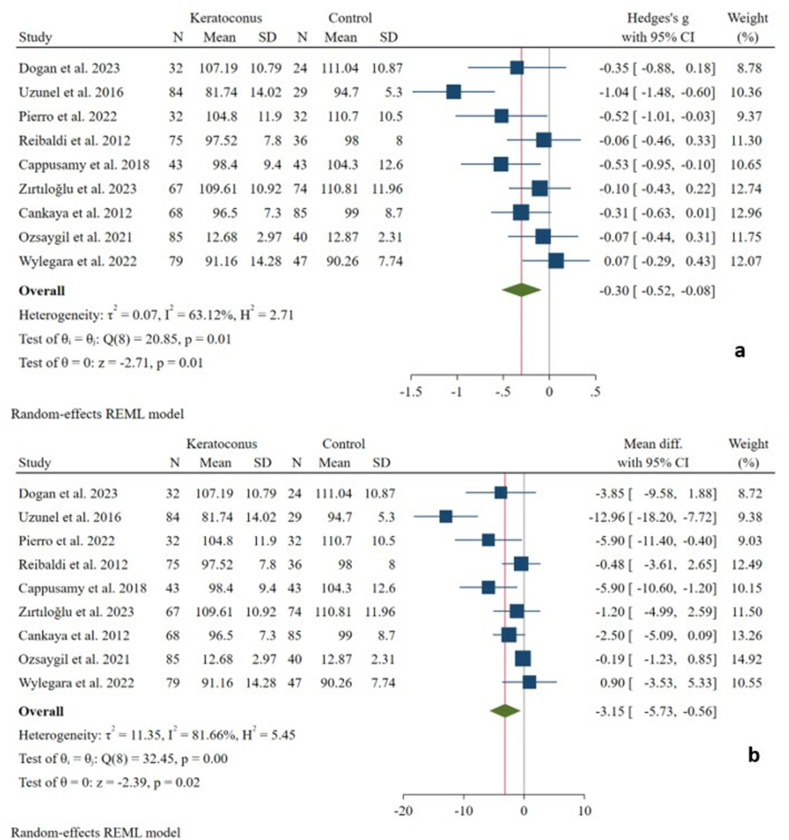
Forest plots of RNFL thickness comparisons in eyes with KC and without KC; pooled Hedge's g with the REML model (a); pooled weighted mean differences (WMD) with the REML model (b).

Nine studies measured the RNFL thickness [1,7,14,15,23,25,27,29,31] in 565 KC eyes and 410 control eyes. When compared to controls, KC eyes had a mildly significant decrease in RNFL thickness (SMD: −0.30 [−0.52, −0.08], I^2^ = 63.1 %) with an absolute reduction of 3.15 μm (WMD: −3.15 [−5.73, −0.56]) (Fig. 2 [a-b]). Subsequent analysis showed that in stage 1, the RNFL thickness had a mildly significant decrease (SMD: −0.35 [−0.63, −0.07], I^2^ = 0.00 %), while there was no significant absolute change (WMD: −1.39 [−3.08, 0.29]). Additionally, there was no significant RNFL thickness difference between the two groups in stages 2 and 3 (Table 3).

The subgroup analysis of RNFL thickness by age groups revealed non significant difference between the two groups (p-value for group difference of SMD = 0.68), and there was a mild significant reduction in RNFL thickness for ages<30 yo (SMD: −0.24 [−0.39, −0.10], I^2^ = 0.00 %), with an absolute reduction of 1.98 μm (WMD: −1.98 [−3.65, −0.31]), while there were no significant changes in ages≥30 yo. Table 4 summarizes the results of the subgroup analysis for measured outcomes (Detailed plots of meta-analyses are shown in Supplementary Figs. S30–S35).

**Table 4:** Subgroup meta-analysis of outcomes^a^.

Outcomes	Subgroup		Hedge's g with 95%CI	P-value (Q)	I^2^ (%)	P-value (group difference)	WMD with 95%CI	P-value (Q)	I^2^ (%)	P-value (group difference)
RNFL	Nationality	Asia	−0.36 [-0.69, −0.02]	0.01	72.99	0.33	−3.75 [-7.94, 0.45]	0.00	89.76	0.29
		Europe	−0.13 [-0.45, 0.19]	0.16	45.25		−1.36 [-4.71, 1.98]	0.15	46.50	
		Africa	−0.53 [-0.95, −0.10]	–	–		−5.90 [-10.60, −1.20]	–	–	
	Quality	Low ROB	−0.29 [-0.70, 0.12]	0.12	57.63	0.94	−3.34 [-7.92, 1.25]	0.13	57.02	0.96
		High ROB	−0.31 [-0.59, −0.03]	0.01	67.91		−3.18 [-6.46, 0.10]	0.00	86.06	
	Age	≥30 yo	−0.47 [-1.56, 0.61]	0.00	93.18	0.68	−5.96 [-19.54, 7.63]	0.00	93.62	0.57
		<30 yo	−0.24 [-0.39, −0.10]	0.47	0.00		−1.98 [-3.65, −0.31]	0.06	48.44	
Foveal macular thickness	Nationality	Asia	0.07 [-0.23, 0.37]	0.00	71.54	0.00	2.20 [-4.38, 8.77]	0.00	75.40	0.00
		North America & Europe	0.09 [-0.19, 0.36]	0.12	47.84		1.9 [-4.06, 7.85]	0.13	47.1	
		Africa	0.28 [-0.06, 0.62]	–	–		5.25 [-1.15, 11.65]	–	–	
		Australia	3.96 [3.57, 4.34]	–	–		10.20 [9.61, 10.79]	–	–	
	Quality	Low ROB	0.63 [-0.32, 1.58]	0.00	98.19	0.20	4.66 [-0.25, 9.57]	0.00	82.84	0.17
		High ROB	−0.02 [-0.31, 0.26]	0.18	37.68		−0.49 [-5.96, 4.98]	0.20	36.8	
	Age	≥30 yo	1.00 [-0.95, 2.94]	0.00	99.08	0.39	3.49 [-3.56, 10.55]	0.00	82.43	0.93
		<30 yo	0.13[-0.10, 0.37]	0.01	64.79		3.10 [-1.98, 8.18]	0.00	69	

a Detailed plots of meta-analyses are shown in Supplementary Figs. S30–S35.

#### 3.4.2 Foveal macular thickness

12 studies measured the foveal macular thickness [1,2,15,[23], [24], [25], [26],^28^,[30], [31], [32], [33]] in 749 KC eyes and 788 control eyes. When compared to controls, there were not any significant changes in foveal macular thickness (Table 3).

The subgroup analysis of foveal macular thickness by age groups, and quality of studies revealed no significant difference between KC eyes and control eyes in each group while the nation sub group analysis reach a significant level (p-value = 0.00) (Table 4).

#### 3.4.3 Parafoveal macular thickness

Three studies assessed the whole parafoveal macular thickness [23,31,33] in 228 KC eyes and 272 control eyes and three studies assessed the sub-regional (superior, temporal, inferior, and nasal) parafoveal macular thickness [2,30,32] in 144 KC eyes and 146 control eyes. There were no statistically significant differences observed in the overall and sub-regional parafoveal macular thickness in comparison to the control group (Table 3).

#### 3.4.4 Perifoveal macular thickness

Three studies evaluated the sub-regional-based perifoveal macular thickness [2,30,32] in 144 KC eyes and 146 control eyes. There was a mildly significant decrease in perifoveal macular thickness in the nasal region (SMD: −0.32 [−0.55, −0.09], I^2^ = 0.00 %) with an absolute reduction of 5.94 μm (WMD: −5.94 [−10.69, −1.19]). Additionally, there was a mildly significant decrease in perifoveal macular thickness in the superior region (SMD: −0.24 [−0.47, −0.01], I^2^ = 0.00 %), while there was no significant absolute change (WMD: −3.80 [−7.32, −0.29]). There were not any statistically significant changes in the other regions (Table 3).

#### 3.4.5 ONH disk area

Four studies measured the ONH disk area [1,2,14,23] in 231 KC eyes and 206 control eyes. There was a mildly to moderately significant increase in ONH disk area (SMD: 0.40 [0.06, 0.75], I^2^ = 67.10 %) with an absolute increase of 0.19 mm^2^ (WMD: 0.19 [0.08, 0.30]) (Table 3).

#### 3.4.6 Superior capillary plexus (SCP)

Three studies evaluated the SCP density [23,25,31] in 131 KC eyes and 130 control eyes. In comparison to controls, there was a severe decrease in SCP density (SMD: −1.52 [−2.27, −0.77], I^2^ = 85.03 %) with an absolute reduction of 4.81 % (WMD: −4.81 [−6.79, −2.83]) (Table 3).

#### 3.4.7 Deep capillary plexus (DCP)

Three studies measured the DCP density [23,25,31] in 131 KC eyes and 130 control eyes. There were not any statistically significant differences in DCP density (Table 3).

#### 3.4.8 GCL thickness

Two studies assessed the GCL thickness [7,15] in 169 KC eyes and 69 control eyes. When compared to controls, there were not any significant differences in overall GCL thickness and between different stages (Table 3).

### 3.5 Sensitivity analysis

**Fig. 3:**
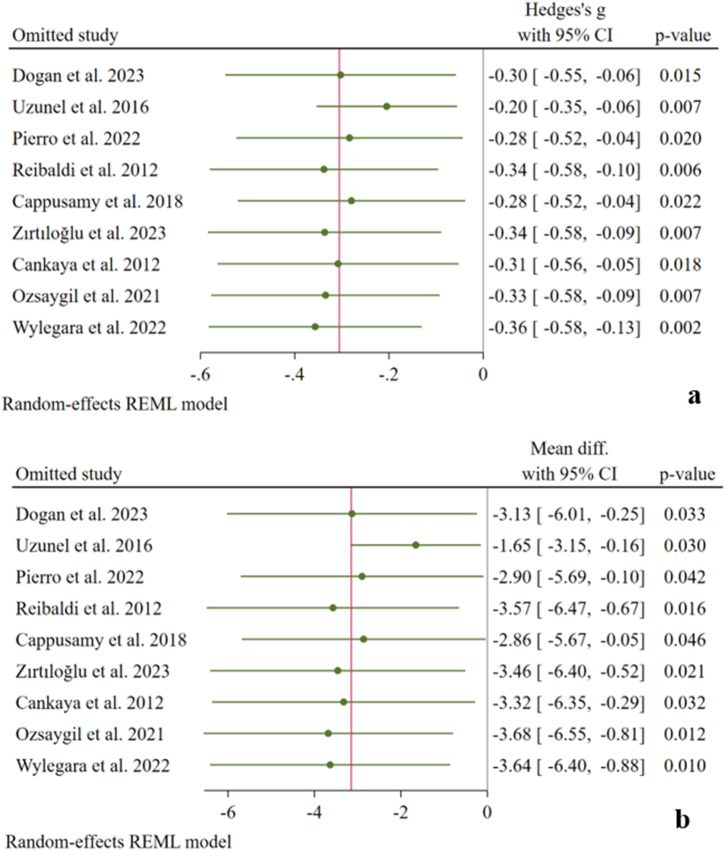
Leave-one-out sensitivity analysis on pooled Hedge's g (a) and weighted mean differences (WMD) (b) of RNFL thickness.

The sensitivity analysis was performed on the RNFL thickness. There was no outlier study to have a significant influence on pooled estimates (Fig. 3 [a-b]).

### 3.6 Publication bias

**Fig. 4:**
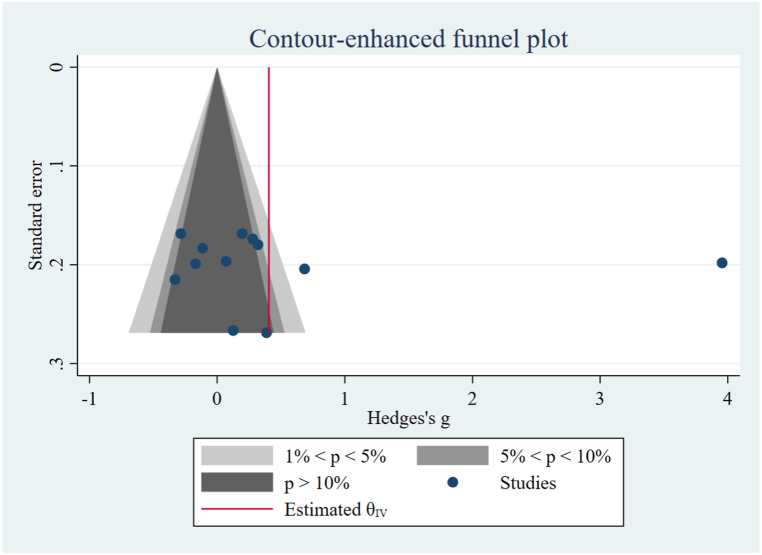
Contour-enhanced funnel plot for foveal macular thickness.

Given the restricted number of studies, we only assessed the contour-enhanced funnel plot for the foveal macular thickness. Our findings indicated an asymmetry raised concerns about the possibility of publication bias for foveal macular thickness (Fig. 4). Nevertheless, Egger's test showed that there was a high risk of publication bias only in the analyses of the RNFL stage 2 (p-value = 0.02) and the macular cube volume (p-value = 0.00) (Table 3). Subsequently, we conducted the trim-and-fill correction process for RNFL stage 2 and macular cube volume, and the meta-analysis results did not considerably change (Supplementary Table S3).

### 3.7 Certainty of the evidence

The GRADE assessment of the certainty of evidence for the analyzed outcomes is summarized in Supplementary Table S4. The overall certainty of the evidence had limited importance for making a decision.

## 4 Discussion

KC, despite common belief, is not a sporadic disease. Previous studies showed that 50 % of KC patients have hyperelastic joints and high incidences of hypersensitivities [34]. KC is also associated with several systematic vascular and connective tissue disorders, such as Ehlers-Danlos syndrome, Marfan syndrome, osteogenesis imperfecta, etc. [1,23,25,31,34]. KC is reported to coexist with both posterior and anterior segment disorders like retinal degeneration, central serous chorioretinopathy, choroidal neovascularization, retinitis pigmentosa, Leber congenital amaurosis, atopic keratoconjunctivitis, and vernal conjunctivitis [23,34]. Since the OCTA introduction, we have had a novel technology that visualizes the retinal microvasculature quickly and non-invasively with a resolution that surpasses fluorescein angiography and comes close to histological accuracy [35].

The current systematic review and meta-analysis identified 16 studies in which OCT/OCTA imaging was used to assess the retinal thickness and vessel density changes in patients with KC. As our main findings, there were considerable changes in certain parts of the retina. Still, due to the low number of included studies and the higher heterogeneity in some outcomes, we should interpret the final measure with caution. Our findings demonstrated a reduction in macular whole thickness, nasal part of perifovea, RNFL thickness in overall patients and stage 1, and GCL of stage 1 KC patients. Additionally, we found a reduction in SCP vessel density and ONH whole vessel density. We also found an increase in the ONH cup area, disk area, and cup volume.

KC was traditionally considered a non-inflammatory disease until Zhou et al. and Pouliquen et al. published articles in 1996, uncovering elevated levels of vimentin, tenascin Transforming growth factor beta (TGF-β), Interleukin-1 (IL-1), and Prostaglandin E2 [36,37]. Their findings paved the way for subsequent studies that identified significantly increased cytokines and inflammatory mediators in both tears and corneal tissue [38]. As mentioned earlier, KC is linked to various allergic disorders such as asthma and atopy, with immunoglobulin E being a common mediator in these conditions. In a study by Kemp et al., total serum immunoglobulin E and serum-specific Immunoglobulin E were measured in KC patients, showing an increase of 52 % and 59 %, respectively [39]. These inflammatory processes facilitate changes to the blood supply, chemical environment, and cellular components of the affected region, potentially serving as a crucial factor in the disruption of retinal and choroidal microvascularity [23,40].

In addition to the inflammatory process, the corneal stroma of KC also exhibits the disarray of collagen lamellae [41]. Given that collagen type 1 is a key component of the corneal stroma, along with the media and adventitia of vascular walls [23,42], and given the documented associations between KC and connective tissue disorders, future studies will need to investigate potential changes in blood vessel size and direct or indirect involvement of the vasculature in KC patients.

Previous studies showed that corneal thickness in glaucomatous patients has a negative correlation with a cup-to-disk ratio and RNFL loss, suggesting that patients with thinner corneas tend to have a larger cup-to-disk ratio and are more susceptible to pressure-induced deformations leading to a greater reduction of RNFL thickness [43,44]. In KC patients, the cornea becomes thinner as the disease progresses and is less effective in filtering out Ultraviolet (UV) radiation, leading to the formation of reactive oxygen species (ROS) [45]. Based on a recently published meta-analysis, an increase in oxidative stress markers, particularly in ROS and nitrogen species, along with a decrease in antioxidants, resulted in a redox hemostasis imbalance of tears, cornea, aqueous humor, and blood in KC patients [46], which could lead to retinal, neural, and vascular damage. Elevated ROS will increase the activation of protein kinase C and NF-κB and increase the formation of advanced glycation end products and sorbitol accumulation, all of which are associated with vascular endothelial cell damage [47]. ROS also impacts the expression of the synaptophysin protein and brain-derived neurotrophic factor (BDNF), which are responsible for synaptic activity, axonal growth, and neuronal survival. Dysregulation in synaptophysin protein and BDNF levels can disrupt neuronal synaptic activity. These changes in synaptic activity can result in neural survival failure by interfering with cell membrane depolarization and increasing intracellular calcium levels [48]. Ali et al. demonstrated that in diabetes, the neural retinal cells are affected by an increase in superoxide anions, which reduces the availability of nitric oxide needed for tissue protection [49]. This decrease in nitric oxide leads to the formation of peroxynitrite, inhibiting the signaling of nerve growth factor (NGF) and impacting neuronal survival. These mechanisms can result in a decrease in retinal layer thickness, making the retina more vulnerable to damage. Consequently, a decrease in macular thickness, particularly the loss of the RNFL accompanied by photoreceptor dysfunction observed in electroretinograms (ERG) [26], along with alterations in retinochoroidal vascular density, leads to impairments in best corrected visual acuity (BCVA) [[50], [51], [52]].

In our meta-analysis, we showed a mild reduction in RNFL thickness compared to normal individuals, and sensitivity analysis revealed the robustness of our findings. To address the moderate statistical heterogeneity (I^2^ = 63.12 %) with subgroup analyses, the continent-based and quality-based analyses did not show significant differences, suggesting that geographical regions or study quality did not impact the results. However, the age-based analysis revealed a significant difference when a cutoff of 30 years old was used, revealing that in younger patients, it was more considerable. Moreover, our analyses showed that these findings were not sensitive to small study effects and could be conclusive.

The considerable reduction in RNFL thickness in patients under 30 years old indicates a higher rate of progression in younger KC patients. Previous studies have also noted this accelerated progression among younger KC patients. We have been diligently monitoring this specific subpopulation, as their vision may deteriorate. However, there is hope that disease progression can be halted or slowed through the use of corneal crosslinking [24,34,53]. Our findings demonstrate that this higher rate of progression is not limited to the cornea, and this subpopulation has been affected by a more flaming phase of the disease [53].

In KC, the supranasal cornea exhibits the greatest average thickness [24,34], while in normal eyes, the RNFL is typically thicker in the superior and inferior quadrants [54]. Our study observed a general reduction in macular thickness, with a significant decrease in the superonasal quadrants. KC is characterized by astigmatism, and there is ongoing debate regarding the influence of astigmatism and refractive errors as potential artifacts in the interpretation of OCT results. Studies have shown that astigmatic defocus, particularly with-the-rule astigmatism, can significantly affect OCT-A metrics, such as vessel density and perfusion density, especially in specific retinal quadrants. This highlights the need to account for these factors in OCT interpretation, as the observed changes may reflect measurement artifacts rather than true physiological alterations in KC patients [55,56]. If these factors are not accounted for as confounders, there may be compensatory mechanisms in response to the altered corneal curvature of KC that warrant further investigation in future studies.

## 5 Limitations and recommendations

The limited sample sizes constrained our ability to conduct more subgroup analyses to explore potential sources of heterogeneity. In particular, the limited number of studies investigating posterior segments at different stages of KC, such as GCL and RNFL thickness, restricts our capacity to draw definitive conclusions. For example, the unexpected decrease in RNFL thickness observed in stage 1 of KC, despite the absence of significant changes in later stages, may be clarified with a larger pool of studies. Expanding the number of studies on these variables would allow for a more robust understanding of their role across different stages of KC and strengthen the certainty of our findings. Also, these smaller sample sizes and weighting method differences could contribute to some discrepancies in SMD and WMD results. To enhance the certainty of the evidence, it is essential to conduct larger and higher-quality primary studies.

To gain the best understanding of retinal changes in KC, future studies should delve into the pathophysiology beyond the involvement of retinal components, such as exploring changes in collagen types 1, 3, and 5, as well as investigating the corneal curvature effect on augmented changes in the retina.

## 6 Conclusion

KC is a multifaceted disease beyond its traditional characterization as a non-inflammatory, sporadic disorder. The advent of OCT/OCTA technology has allowed for detailed visualization of retinal changes in KC, showing reductions in macular thickness, RNFL thickness, and vessel density, particularly in younger patients. While these findings do not currently alter treatment approaches, they provide valuable insights into the retinal involvement in KC. Understanding these changes could be crucial for future research, potentially aiding in the development of early detection methods or prognostic indicators that further illuminate the retina-corneal relationship in KC patients. This comprehensive review and meta-analysis reveal that KC patients exhibit retinal abnormalities in addition to various systemic and ocular comorbidities. While it is not yet clear if these changes precede or are concomitant with KC, it is important to note that low visual acuity in KC is not solely attributable to corneal abnormalities. Although rigid contact lenses can correct most of the decreased visual acuity by addressing corneal irregularities, the potential impact of retinal involvement cannot be entirely ruled out, as it may have implications for visual function that extend beyond corneal factors. Additionally, the interplay between inflammatory processes, oxidative stress, and vascular changes underscores the complex pathophysiology of KC which requires future research to focus on further elucidating these mechanisms.

## CRediT authorship contribution statement

**Hadi Vahedi:** Writing – original draft, Validation, Investigation, Data curation, Conceptualization. **Mirsaeed Abdollahi:** Writing – original draft, Methodology, Formal analysis, Data curation. **Reza Moshfeghinia:** Writing – original draft, Formal analysis. **Shima Emami:** Writing – original draft, Investigation. **Navid Sobhi:** Writing – review & editing, Visualization, Validation. **Rana Sorkhabi:** Writing – review & editing, Supervision. **Ali Jafarizadeh:** Writing – review & editing, Supervision, Project administration, Methodology, Conceptualization.

## Ethics approval and consent to participate

Not applicable.

## Consent for publication

Not applicable.

## Data availability statement

All data is provided within the manuscript and supporting information files. For any additional inquiries, please contact the corresponding authors.

## Funding

None.

## Declaration of competing interest

The authors declare that they have no known competing financial interests or personal relationships that could have appeared to influence the work reported in this paper.
