# Supplementary material for: Retinal thickness and vascular density changes in Keratoconus: A systematic review and meta-analysis
Source: Heliyon. 2025 Jan 18;11(2):e42099. doi: 10.1016/j.heliyon.2025.e42099 (PMC11804566; doi:10.1016/j.heliyon.2025.e42099)
Supplement: Multimedia component 1 [file mmc1.docx]

**Supplementary Tables**

Supplementary Table S1 - Search syntax

| Database | Search Syntax | Results |
| --- | --- | --- |
| PubMed | (((keratoconus) OR (kcn)) AND ((optical coherence tomography) OR (optical coherence tomography angiography) OR (OCTA) OR (retinal vessel density) OR (retinal vascular density) OR (foveal avascular zone) OR (central foveal thickness) OR (parafoveal thickness) OR (perifoveal thickness) OR (macular thickness) OR (retinal nerve fiber layer) OR (RNFL) OR (retinal layer) OR (retinal pigment epithelium) OR (retinal ganglion cell) OR (disk area) OR (rim area) OR (cup volume) OR (cube volume)))  ("keratoconus"[MeSH Terms] OR "keratoconus"[All Fields] OR "kcn"[All Fields]) AND ("tomography, optical coherence"[MeSH Terms] OR ("tomography"[All Fields] AND "optical"[All Fields] AND "coherence"[All Fields]) OR "optical coherence tomography"[All Fields] OR ("optical"[All Fields] AND "coherence"[All Fields] AND "tomography"[All Fields]) OR (("tomography, optical coherence"[MeSH Terms] OR ("tomography"[All Fields] AND "optical"[All Fields] AND "coherence"[All Fields]) OR "optical coherence tomography"[All Fields] OR ("optical"[All Fields] AND "coherence"[All Fields] AND "tomography"[All Fields])) AND ("angiography"[MeSH Terms] OR "angiography"[All Fields] OR "angiographies"[All Fields] OR "angiography s"[All Fields])) OR "OCTA"[All Fields] OR (("retinal vessels"[MeSH Terms] OR ("retinal"[All Fields] AND "vessels"[All Fields]) OR "retinal vessels"[All Fields] OR ("retinal"[All Fields] AND "vessel"[All Fields]) OR "retinal vessel"[All Fields]) AND ("densities"[All Fields] OR "density"[All Fields])) OR (("retinaldehyde"[MeSH Terms] OR "retinaldehyde"[All Fields] OR "retinal"[All Fields] OR "retina"[MeSH Terms] OR "retina"[All Fields] OR "retinally"[All Fields] OR "retinals"[All Fields] OR "retinitis"[MeSH Terms] OR "retinitis"[All Fields]) AND ("microvascular density"[MeSH Terms] OR ("microvascular"[All Fields] AND "density"[All Fields]) OR "microvascular density"[All Fields] OR ("vascular"[All Fields] AND "density"[All Fields]) OR "vascular density"[All Fields])) OR (("foveal"[All Fields] OR "foveally"[All Fields]) AND ("avascular"[All Fields] OR "avascularity"[All Fields] OR "avascularized"[All Fields]) AND "zone"[All Fields]) OR (("central"[All Fields] OR "centrally"[All Fields] OR "centrals"[All Fields]) AND ("foveal"[All Fields] OR "foveally"[All Fields]) AND ("thick"[All Fields] OR "thickness"[All Fields] OR "thicknesses"[All Fields])) OR (("parafoveal"[All Fields] OR "parafoveally"[All Fields]) AND ("thick"[All Fields] OR "thickness"[All Fields] OR "thicknesses"[All Fields])) OR ("perifoveal"[All Fields] AND ("thick"[All Fields] OR "thickness"[All Fields] OR "thicknesses"[All Fields])) OR ("macular"[All Fields] AND ("thick"[All Fields] OR "thickness"[All Fields] OR "thicknesses"[All Fields])) OR (("retinaldehyde"[MeSH Terms] OR "retinaldehyde"[All Fields] OR "retinal"[All Fields] OR "retina"[MeSH Terms] OR "retina"[All Fields] OR "retinally"[All Fields] OR "retinals"[All Fields] OR "retinitis"[MeSH Terms] OR "retinitis"[All Fields]) AND ("nerve fibre"[All Fields] OR "nerve fibers"[MeSH Terms] OR ("nerve"[All Fields] AND "fibers"[All Fields]) OR "nerve fibers"[All Fields] OR ("nerve"[All Fields] AND "fiber"[All Fields]) OR "nerve fiber"[All Fields]) AND ("layer"[All Fields] OR "layer s"[All Fields] OR "layered"[All Fields] OR "layering"[All Fields] OR "layerings"[All Fields] OR "layers"[All Fields])) OR "RNFL"[All Fields] OR (("retinaldehyde"[MeSH Terms] OR "retinaldehyde"[All Fields] OR "retinal"[All Fields] OR "retina"[MeSH Terms] OR "retina"[All Fields] OR "retinally"[All Fields] OR "retinals"[All Fields] OR "retinitis"[MeSH Terms] OR "retinitis"[All Fields]) AND ("layer"[All Fields] OR "layer s"[All Fields] OR "layered"[All Fields] OR "layering"[All Fields] OR "layerings"[All Fields] OR "layers"[All Fields])) OR ("retinal pigment epithelium"[MeSH Terms] OR ("retinal"[All Fields] AND "pigment"[All Fields] AND "epithelium"[All Fields]) OR "retinal pigment epithelium"[All Fields]) OR ("retinal ganglion cells"[MeSH Terms] OR ("retinal"[All Fields] AND "ganglion"[All Fields] AND "cells"[All Fields]) OR "retinal ganglion cells"[All Fields] OR ("retinal"[All Fields] AND "ganglion"[All Fields] AND "cell"[All Fields]) OR "retinal ganglion cell"[All Fields]) OR ("disk"[All Fields] AND ("geographic locations"[MeSH Terms] OR ("geographic"[All Fields] AND "locations"[All Fields]) OR "geographic locations"[All Fields] OR "area"[All Fields])) OR ("rim"[All Fields] AND ("geographic locations"[MeSH Terms] OR ("geographic"[All Fields] AND "locations"[All Fields]) OR "geographic locations"[All Fields] OR "area"[All Fields])) OR ("cup"[All Fields] AND ("volum"[All Fields] OR "volume"[All Fields] OR "volumes"[All Fields] OR "voluming"[All Fields])) OR ("cube"[All Fields] AND ("volum"[All Fields] OR "volume"[All Fields] OR "volumes"[All Fields] OR "voluming"[All Fields]))) | **823** |
| Scopus | TITLE-ABS-KEY ( ( "keratoconus" OR "kcn" ) AND ( "optical coherence tomography" OR "optical coherence tomography angiography" OR "OCTA" OR "retinal vessel density" OR "retinal vascular density" OR "foveal avascular zone" OR "central foveal thickness" OR "parafoveal thickness" OR "perifoveal thickness" OR "macular thickness" OR "retinal nerve fiber layer" OR "RNFL" OR "retinal layer" OR "retinal pigment epithelium" OR "retinal ganglion cell" OR "disk area" OR "rim area" OR "cup volume" OR "cube volume" ) ) | **1387** |
| Embase | ('keratoconus' OR 'kcn') AND ('optical coherence tomography' OR 'optical coherence tomography angiography' OR 'octa' OR 'retinal vessel density' OR 'retinal vascular density' OR 'foveal avascular zone' OR 'central foveal thickness' OR 'parafoveal thickness' OR 'perifoveal thickness' OR 'macular thickness' OR 'retinal nerve fiber layer' OR 'rnfl' OR 'retinal layer' OR 'retinal pigment epithelium' OR 'retinal ganglion cell' OR 'disk area' OR 'rim area' OR 'cup volume' OR 'cube volume') | **1431** |
| Web of Science | ((ALL=(keratoconus) OR ALL=(KCN)) AND (ALL=(optical coherence tomography) OR ALL=(optical coherence tomography angiography) OR ALL=(OCTA) OR ALL=(retinal vessel density) OR ALL=(retinal vascular density) OR ALL=(foveal avascular zone) OR ALL=(central foveal thickness) OR ALL=(perifovea thickness) OR ALL=(parafoveal thickness) OR ALL=(macular thickness) OR ALL=(retinal nerve fiber layer) OR ALL=(RNFL) OR ALL=(retinal layer) OR ALL=(retinal pigment epithelium) OR ALL=(retinal ganglion cell) OR ALL=(disk area) OR ALL=(rim area) OR ALL=(cup volume) OR ALL=(cube volume))) | **967** |

Supplementary Table S2 - Excluded studies after full-text assessment

| Reason for exclusion | Article Name | Authors | # |
| --- | --- | --- | --- |
| Review article | Optical coherence tomography for the investigation of posterior and anterior segments | Yoshiaki Yasuno | **1** |
| Irrelevant PECO (intervention: AS-OCT) | Conventional and Iontophoresis Corneal Cross-Linking for Keratoconus: Efficacy and Assessment by Optical Coherence Tomography and Confocal Microscopy | Léa Jouve | **2** |
| Irrelevant PECO (outcome: retinal vascular caliber, Not used OCT) | Alterations in the choroidal thickness and retinal vascular caliber in keratoconus | Gözde Aksoy Aydemir | **3** |
| Irrelevant PECO (intervention: different type of OCT protocol) | Posterior pole retinal thickness distribution pattern in keratoconus | Ali Mahdavi Fard | **4** |
| Unsuitable Study design | Changes in retinal vessel and retinal layer thickness after cross-linking in keratoconus via swept‑source OCT angiography | Yusuf Ayaz | **5** |
| Unsuitable Study design | Macular parameters with and without scleral contact lens in keratoconus using Spectralis optical coherence tomography | João Pinheiro-Costa | **6** |
| Unsuitable Study design | Comparison of Macular Thickness Measured by Optical Coherence Tomography After Deep Anterior Lamellar Keratoplasty and Penetrating Keratoplasty | Banu Torun Acar | **7** |
| Unsuitable Study design | Changes in Central Macular Thickness After Uncomplicated Corneal Transplantation for Keratoconus: Penetrating Keratoplasty Versus Deep Anterior Lamellar Keratoplasty | Arif Koytak | **8** |
| Unsuitable Study design | Effects of rigid contact lenses on optical coherence tomographic parameters in eyes with keratoconus | Umut Duygu Uzunel | **9** |

Supplementary Table S3 - Trim and fill test results on the pooled RNFL stage 2 and macular cube volume

| Studies | **RNFL stage 2**  **Hedges’ g with 95%CI** | **Macular cube volume**  **Hedges’ g with 95%CI** |
| --- | --- | --- |
| Observed | -0.24 [-1.05, 0.57] | -0.11 [-0.46, 0.24] |
| Observed + Imputed | -0.24 [-1.05, 0.57] | -0.11 [-0.46, 0.24] |

Supplementary Table S4 - Grading of Recommendations, Assessment, Development, and Evaluations

| **Outcomes** | **Quality of evidence** | **Risk of bias** | **Inconsistency** | **Indirectness** | **Imprecision** | **Small study effect** | **Justification for Upgrading** | **Certainty** | **Effects** |
| --- | --- | --- | --- | --- | --- | --- | --- | --- | --- |
|  |  |  |  |  |  |  |  |  | **SMD (95% CI)** |
| Macular whole thickness | ꚚOOO Very Low | Low | No (0) | No serious indirectness | Serious (-2) | Undetected (0) | No (0) | Limited importance for making a decision | -0.34 [-0.64, -0.04] |
| Foveal macular thickness | ꚚOOO Very Low | Low | Serious (-2) | No serious indirectness | No (0) | Undetected (0) | No (0) | Limited importance for making a decision | 0.43 [-0.23, 1.08] |
| Parafoveal macular thickness (overall) | ꚚOOO Very Low | Low | Serious (-2) | No serious indirectness | Serious (-2) | Undetected (0) | Upgraded (+2) for large effect size | Limited importance for making a decision | 1.79 [-2.10, 5.68] |
| Parafoveal macular thickness (inferior) | ꚚOOO Very Low | Low | No (0) | No serious indirectness | Serious (-2) | Undetected (0) | No (0) | Limited importance for making a decision | -0.07 [-0.30, 0.15] |
| Parafoveal macular thickness (nasal) | ꚚOOO Very Low | Low | No (0) | No serious indirectness | Serious (-2) | Undetected (0) | No (0) | Limited importance for making a decision | -0.10 [-0.33, 0.13] |
| Parafoveal macular thickness (superior) | ꚚOOO Very Low | Low | No (0) | No serious indirectness | Serious (-2) | Undetected (0) | No (0) | Limited importance for making a decision | -0.12 [-0.34, 0.11] |
| Parafoveal macular thickness (temporal) | ꚚOOO Very Low | Low | No (0) | No serious indirectness | Serious (-2) | Undetected (0) | No (0) | Limited importance for making a decision | -0.05 [-0.27, 0.18] |
| Perifoveal macular thickness (overall) | ꚚOOO Very Low | Low | No (0) | No serious indirectness | Serious (-2) | Undetected (0) | No (0) | Limited importance for making a decision | 2.96 [-1.04, 6.96] |
| Perifoveal macular thickness (inferior) | ꚚOOO Very Low | Low | No (0) | No serious indirectness | Serious (-2) | Undetected (0) | No (0) | Limited importance for making a decision | -0.09 [-0.32, 0.14] |
| Perifoveal macular thickness (nasal) | ꚚOOO Very Low | Low | No (0) | No serious indirectness | Serious (-2) | Undetected (0) | No (0) | Limited importance for making a decision | -0.32 [-0.55, -0.09] |
| Perifoveal macular thickness (superior) | ꚚOOO Very Low | Low | No (0) | No serious indirectness | Serious (-2) | Undetected (0) | No (0) | Limited importance for making a decision | -0.24 [-0.47, -0.01] |
| Perifoveal macular thickness (temporal) | ꚚOOO Very Low | Low | No (0) | No serious indirectness | Serious (-2) | Undetected (0) | No (0) | Limited importance for making a decision | -0.12 [-0.38, 0.14] |
| RNFL (overall) | ꚚOOO Very Low | Moderate | Moderate (-1) | No serious indirectness | No (0) | Undetected (0) | No (0) | Limited importance for making a decision | -0.30 [-0.52, -0.08] |
| RNFL stage 1 | ꚚOOO Very Low | Moderate | No (0) | No serious indirectness | Serious (-2) | Undetected (0) | No (0) | Limited importance for making a decision | -0.35 [-0.63, -0.07] |
| RNFL stage 2 | ꚚOOO Very Low | Moderate | Serious (-2) | No serious indirectness | Serious (-2) | moderate (-1) | No (0) | Limited importance for making a decision | -0.24 [-1.06, 0.58] |
| RNFL stage 3 | ꚚOOO Very Low | Moderate | Serious (-2) | No serious indirectness | Serious (-2) | Undetected (0) | Upgraded (+1) for moderate effect size | Limited importance for making a decision | -0.72 [-1.88, 0.44] |
| GCL (overall) | ꚚOOO Very Low | Moderate | Serious (-2) | No serious indirectness | Serious (-2) | Undetected (0) | Upgraded (+1) for moderate effect size | Limited importance for making a decision | -0.56 [-1.40, 0.27] |
| GCL stage 1 | ꚚOOO Very Low | Moderate | No (0) | No serious indirectness | Serious (-2) | Undetected (0) | No (0) | Limited importance for making a decision | -0.47 [-1.02, 0.07] |
| GCL stage 2 | ꚚOOO Very Low | Moderate | Serious (-2) | No serious indirectness | Serious (-2) | Undetected (0) | No (0) | Limited importance for making a decision | -0.47 [-1.35, 0.41] |
| GCL stage 3 | ꚚOOO Very Low | Moderate | Serious (-2) | No serious indirectness | Serious (-2) | Undetected (0) | Upgraded (+2) for large effect size | Limited importance for making a decision | -0.83 [-2.2, 0.54] |
| ONH cup area | ꚚOOO Very Low | Low | No (0) | No serious indirectness | Serious (-2) | Undetected (0) | No (0) | Limited importance for making a decision | 0.36 [0.11, 0.61] |
| ONH disk area | ꚚOOO Very Low | Moderate | Moderate (-1) | No serious indirectness | Moderate (-1) | Undetected (0) | No (0) | Limited importance for making a decision | 0.40 [0.06, 0.75] |
| ONH rim area | ꚚOOO Very Low | Moderate | Moderate (-1) | No serious indirectness | Serious (-2) | Undetected (0) | No (0) | Limited importance for making a decision | 0.28 [-0.10, 0.66] |
| ONH cup volume | ꚚOOO Very Low | Moderate | No (0) | No serious indirectness | Serious (-2) | Undetected (0) | No (0) | Limited importance for making a decision | 0.24 [0.01, 0.48] |
| Macular cube volume | ꚚOOO Very Low | Low | Moderate (-1) | No serious indirectness | Moderate (-1) | moderate (-1) | No (0) | Limited importance for making a decision | -0.11 [-0.46, 0.24] |
| Macular whole vessel density SCP | ꚚOOO Very Low | Moderate | Serious (-2) | No serious indirectness | Serious (-2) | Undetected (0) | Upgraded (+2) for large effect size | Limited importance for making a decision | -1.52[-2.27, -0.77] |
| Macular whole vessel density DCP | ꚚOOO Very Low | Moderate | Serious (-2) | No serious indirectness | Serious (-2) | Undetected (0) | Upgraded (+2) for large effect size | Limited importance for making a decision | -0.96 [-2.57, 0.65] |
| ONH whole vessel density | ꚚOOO Very Low | Low | No (0) | No serious indirectness | Serious (-2) | Undetected (0) | Upgraded (+1) for moderate effect size | Limited importance for making a decision | -0.52 [-0.80, -0.23] |
| ONH peripapillary vessel density | ꚚOOO Very Low | Moderate | Serious (-2) | No serious indirectness | Serious (-2) | Undetected (0) | No (0) | Limited importance for making a decision | 0.14 [-0.40, 0.67] |
| FAZ area | ꚚOOO Very Low | Moderate | No (0) | No serious indirectness | Serious (-2) | Undetected (0) | No (0) | Limited importance for making a decision | -0.27 [-0.70, 0.17] |

**Supplementary Figures**


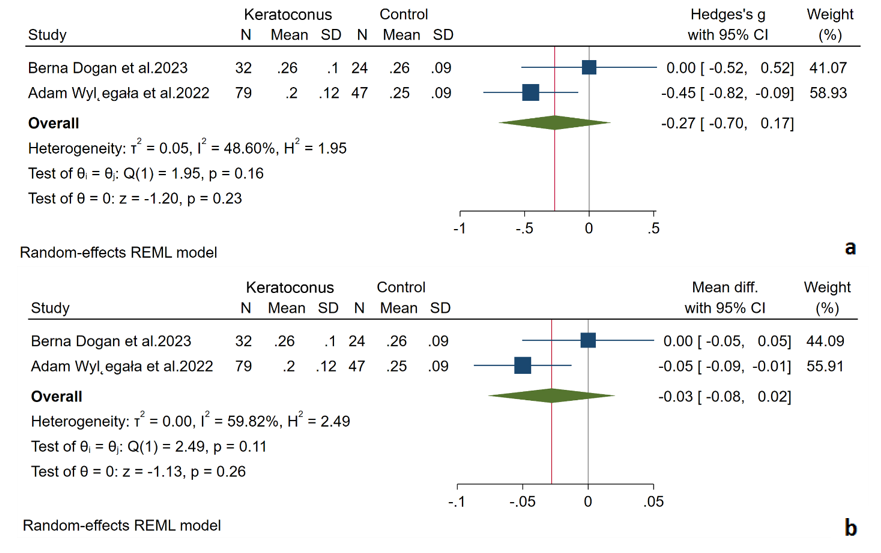


Supplementary Figure S1 - Forest plots of FAZ area comparisons in eyes with KC and without KC; pooled Hedge's g with the REML model (**a**); pooled weighted mean differences (WMD) with the REML model (**b**)


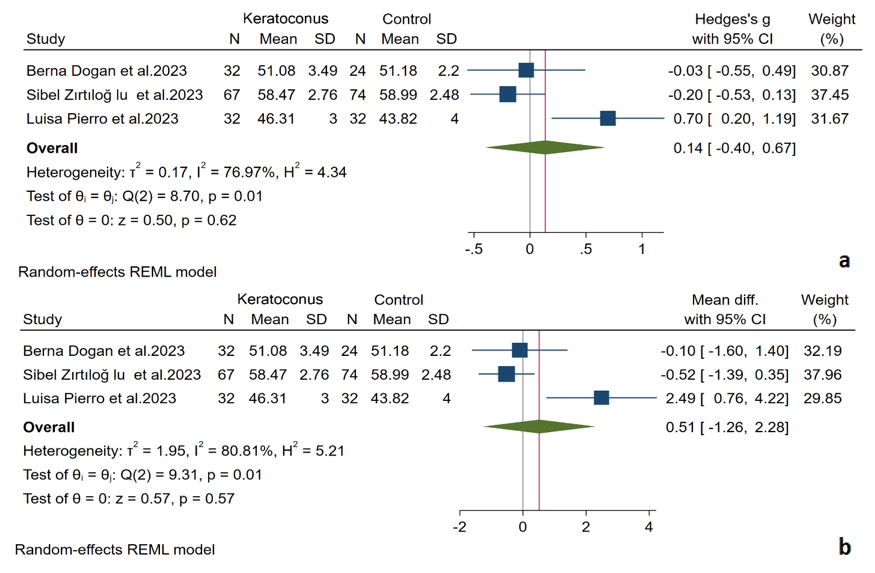
Supplementary Figure S2 - Forest plots of ONH peripapillary vessel density comparisons in eyes with KC and without KC; pooled Hedge's g with the REML model (**a**); pooled weighted mean differences (WMD) with the REML model (**b**)


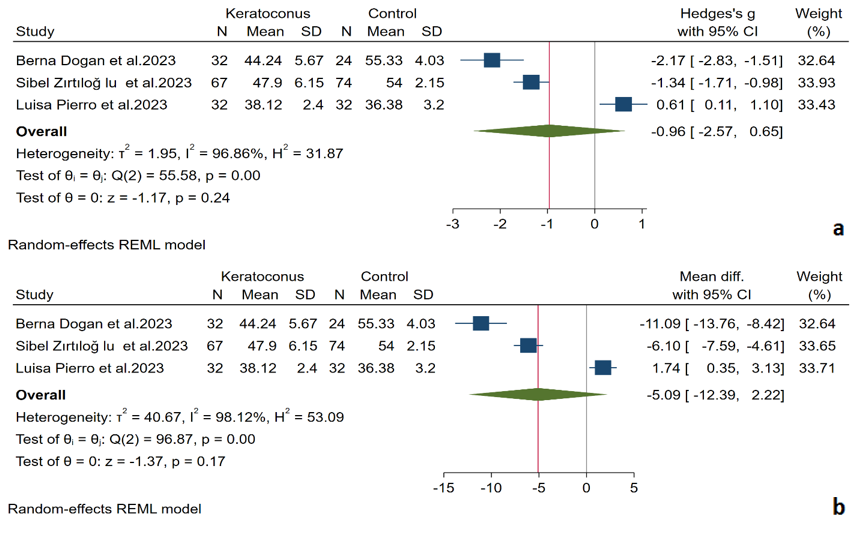
Supplementary Figure S3 - Forest plots of Macular whole vessel density DCP comparisons in eyes with KC and without KC; pooled Hedge's g with the REML model (**a**); pooled weighted mean differences (WMD) with the REML model (**b**)


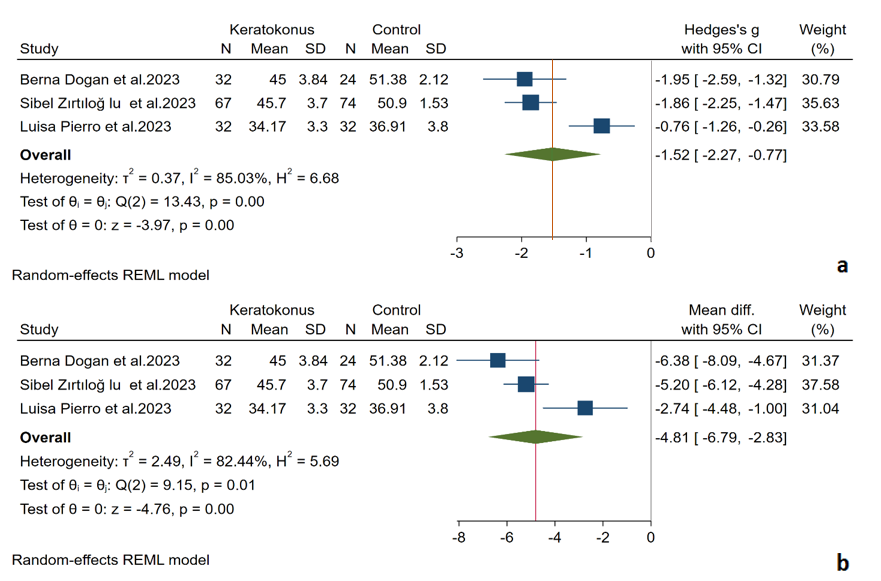
Supplementary Figure S4 - Forest plots of Macular whole vessel density SCP comparisons in eyes with KC and without KC; pooled Hedge's g with the REML model (**a**); pooled weighted mean differences (WMD) with the REML model (**b**)


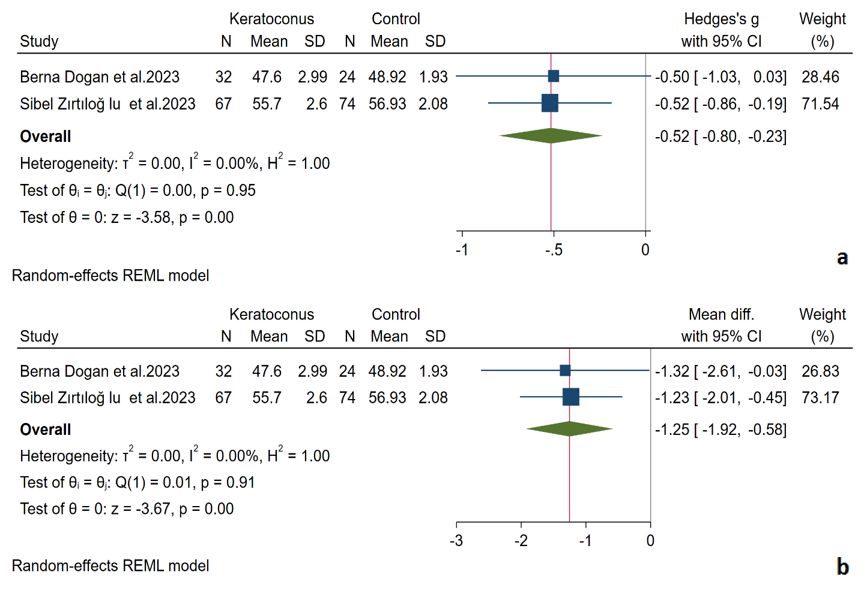
Supplementary Figure S5 - Forest plots of ONH whole vessel density comparisons in eyes with KC and without KC; pooled Hedge's g with the REML model (**a**); pooled weighted mean differences (WMD) with the REML model (**b**)


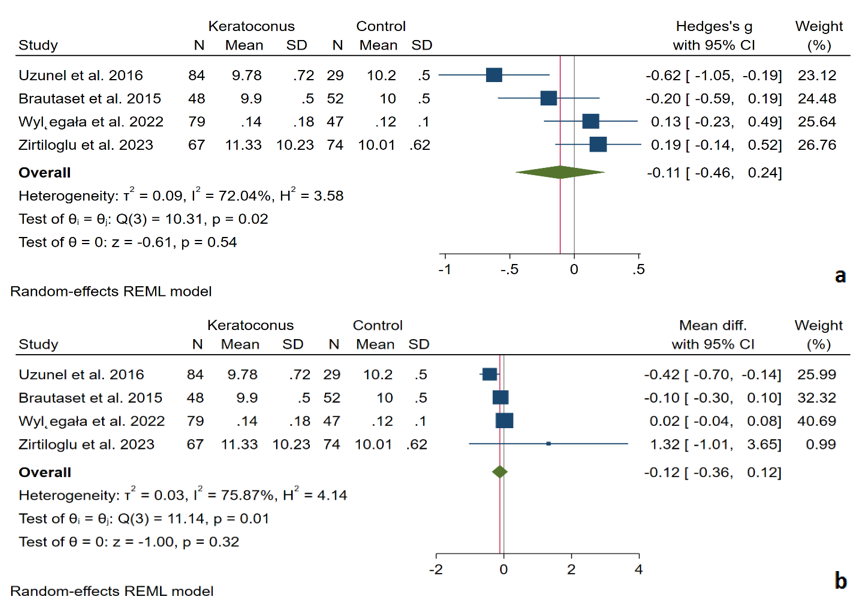


Supplementary Figure S6 - Forest plots of Macular cube volume comparisons in eyes with KC and without KC; pooled Hedge's g with the REML model (**a**); pooled weighted mean differences (WMD) with the REML model (**b**)


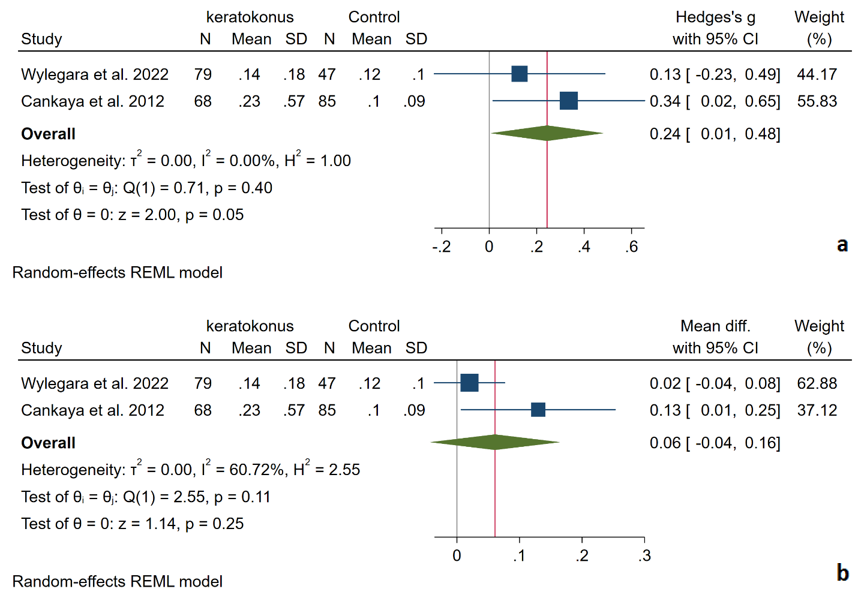
Supplementary Figure S7 - Forest plots of ONH cup volume comparisons in eyes with KC and without KC; pooled Hedge's g with the REML model (**a**); pooled weighted mean differences (WMD) with the REML model (**b**)


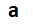

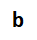

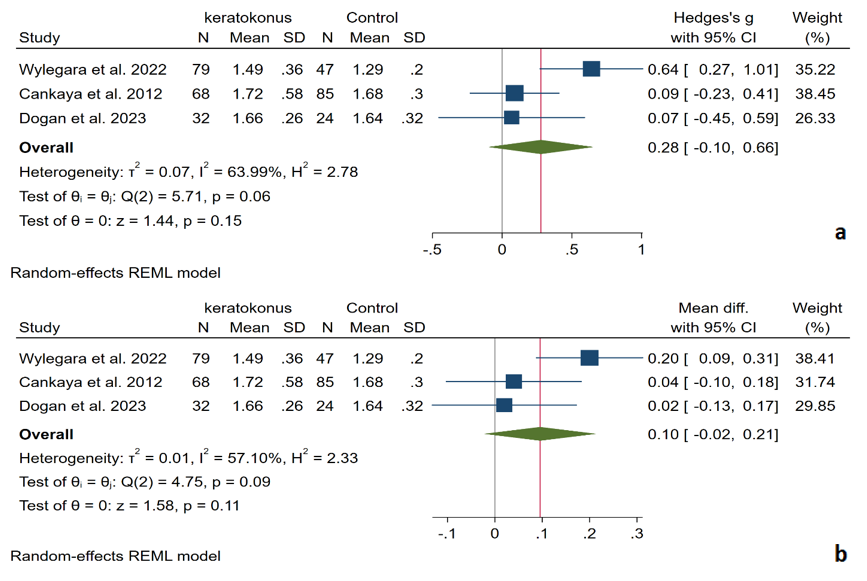
Supplementary Figure S8 - Forest plots of ONH rim area comparisons in eyes with KC and without KC; pooled Hedge's g with the REML model (**a**); pooled weighted mean differences (WMD) with the REML model (**b**)


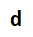

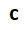


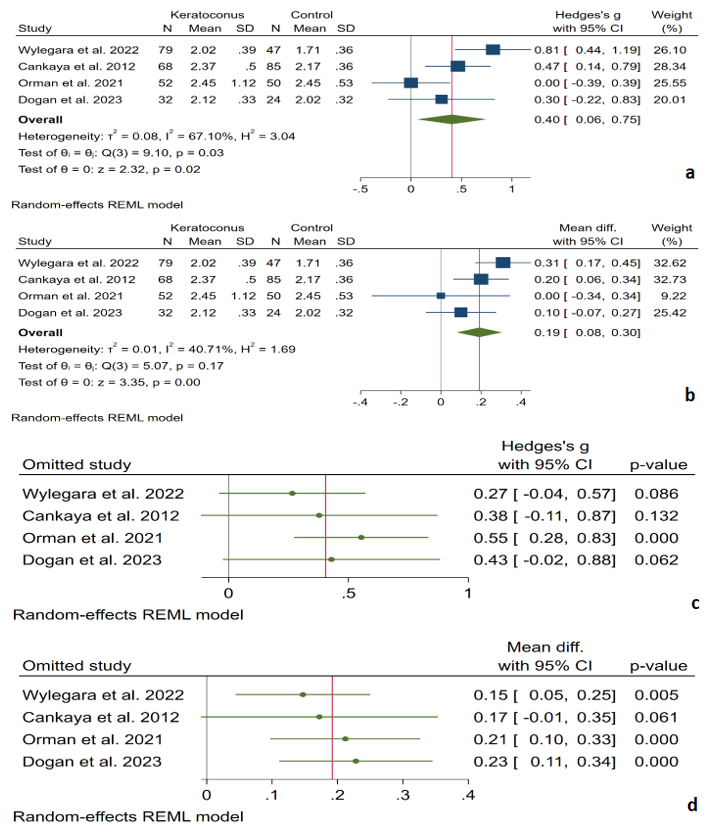
Supplementary Figure S9 - Forest plots of ONH disk area comparisons in eyes with KC and without KC; pooled Hedge's g with the REML model (**a**); pooled weighted mean differences (WMD) with the REML model (**b**) Leave-one-out sensitivity analysis on pooled Hedge's g (**c**) and weighted mean differences (WMD) (**d**) of ONH disk area


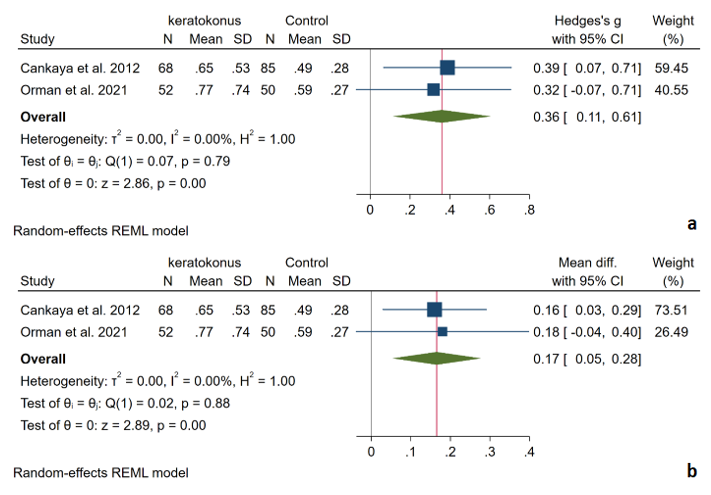
Supplementary Figure S10 - Forest plots of ONH cup area comparisons in eyes with KC and without KC; pooled Hedge's g with the REML model (**a**); pooled weighted mean differences (WMD) with the REML model (**b**)


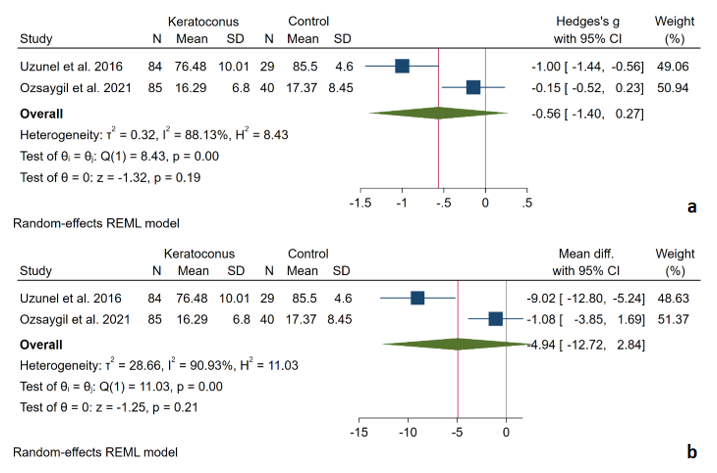
Supplementary Figure S11 - Forest plots of GCL comparisons in eyes with KC and without KC; pooled Hedge's g with the REML model (**a**); pooled weighted mean differences (WMD) with the REML model (**b**)


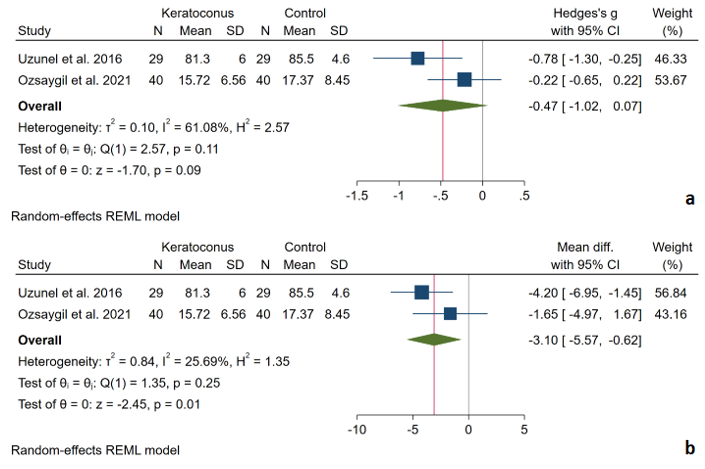
Supplementary Figure S12 - Forest plots of GCL stage 1 comparisons in eyes with KC and without KC; pooled Hedge's g with the REML model (**a**); pooled weighted mean differences (WMD) with the REML model (**b**)


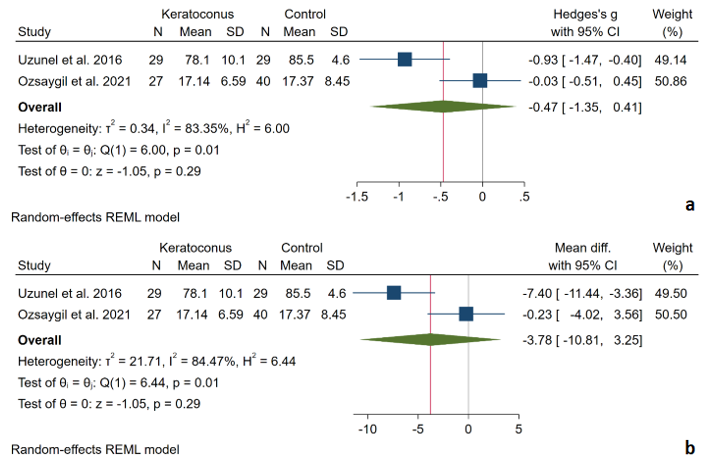
Supplementary Figure S13 - Forest plots of GCL stage 2 comparisons in eyes with KC and without KC; pooled Hedge's g with the REML model (**a**); pooled weighted mean differences (WMD) with the REML model (**b**)


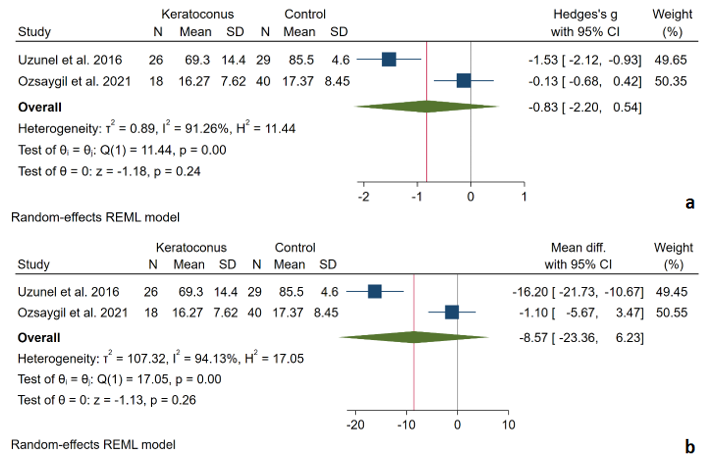


Supplementary Figure S14 - Forest plots of GCL stage 3 comparisons in eyes with KC and without KC; pooled Hedge's g with the REML model (**a**); pooled weighted mean differences (WMD) with the REML model (**b**)


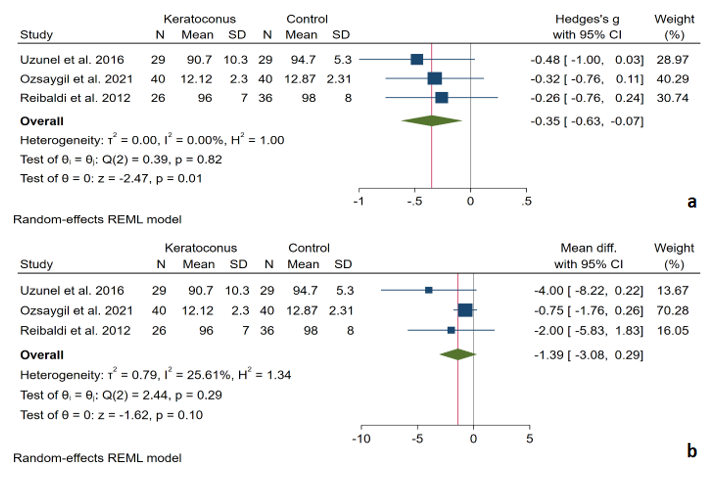


Supplementary Figure S15 - Forest plots of RNFL stage 1 comparisons in eyes with KC and without KC; pooled Hedge's g with the REML model (**a**); pooled weighted mean differences (WMD) with the REML model (**b**)


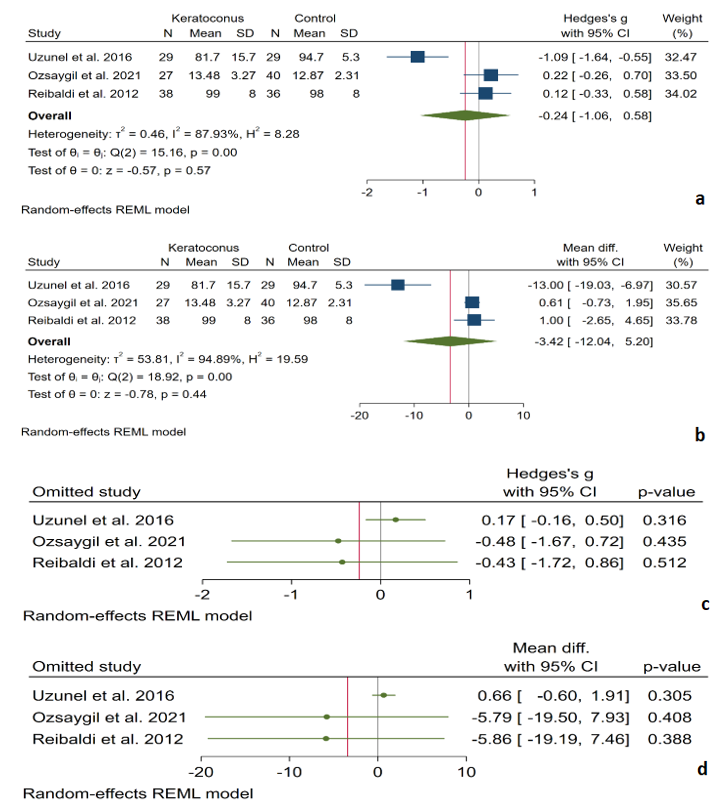


Supplementary Figure S16 - Forest plots of RNFL stage 2 comparisons in eyes with KC and without KC; pooled Hedge's g with the REML model (**a**); pooled weighted mean differences (WMD) with the REML model (**b**) Leave-one-out sensitivity analysis on pooled Hedge's g (**c**) and weighted mean differences (WMD) (**d**) of RNFL stage 2


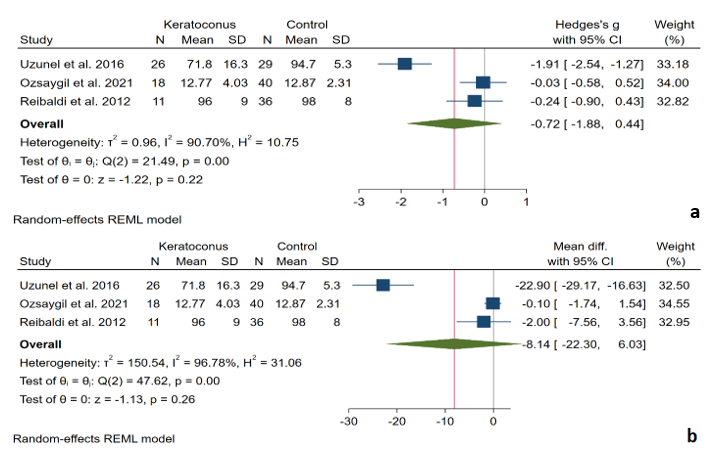


Supplementary Figure S17 - Forest plots of RNFL stage 3 comparisons in eyes with KC and without KC; pooled Hedge's g with the REML model (**a**); pooled weighted mean differences (WMD) with the REML model (**b**)


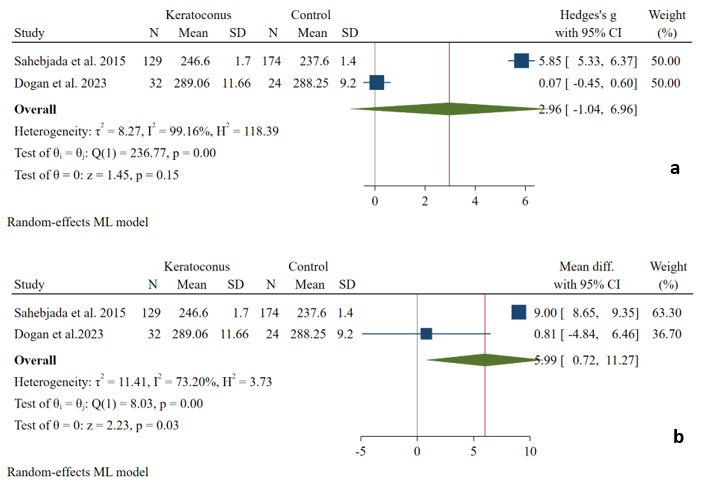


Supplementary Figure S18 - Forest plots of perifovea macular thickness (overall) comparisons in eyes with KC and without KC; pooled Hedge's g with the ML model (**a**); pooled weighted mean differences (WMD) with the REML model (**b**)


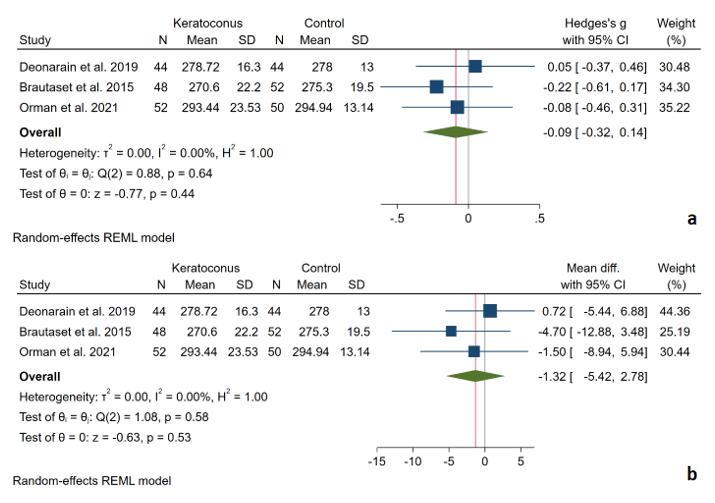


Supplementary Figure S19 - Forest plots of perifoveal macular thickness (inferior) comparisons in eyes with KC and without KC; pooled Hedge's g with the REML model (**a**); pooled weighted mean differences (WMD) with the REML model (**b**)


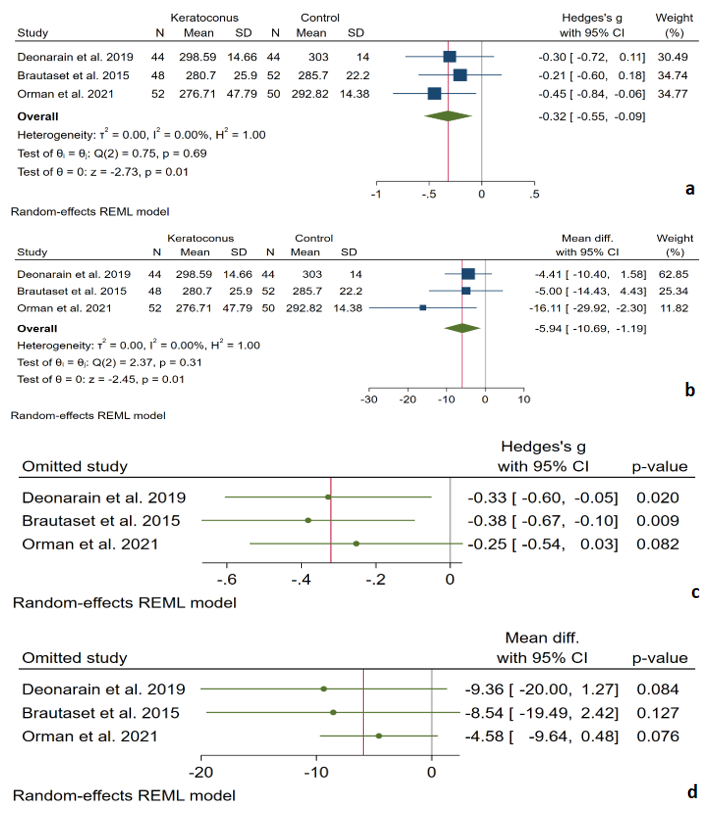
Supplementary Figure S20 - Forest plots of perifoveal macular thickness (nasal) comparisons in eyes with KC and without KC; pooled Hedge's g with the REML model (**a**); pooled weighted mean differences (WMD) with the REML model (**b**) Leave-one-out sensitivity analysis on pooled Hedge's g (**c**) and weighted mean differences (WMD) (**d**) of perifoveal macular thickness (nasal)


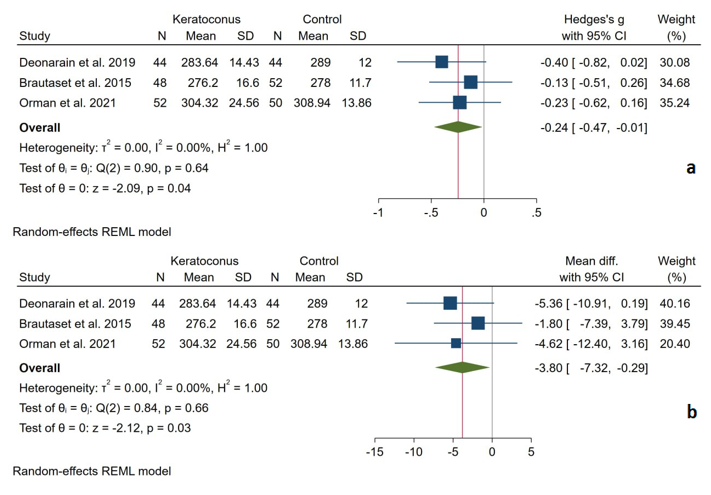
Supplementary Figure S21 - Forest plots of perifoveal macular thickness (superior) comparisons in eyes with KC and without KC; pooled Hedge's g with the REML model (**a**); pooled weighted mean differences (WMD) with the REML model (**b**)


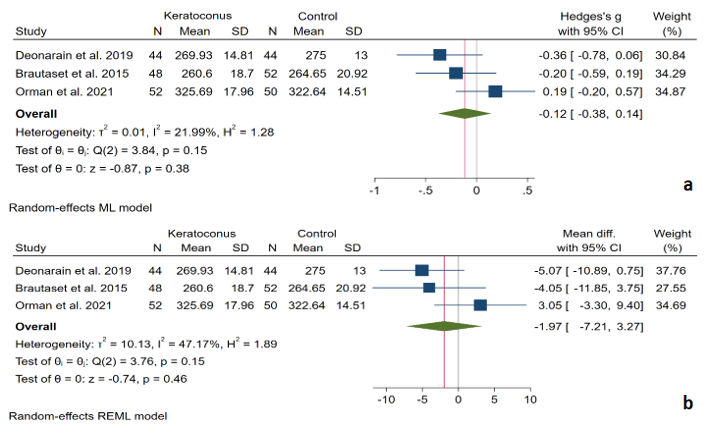
Supplementary Figure S22 - Forest plots of perifoveal macular thickness (temporal) comparisons in eyes with KC and without KC; pooled Hedge's g with the ML model (**a**); pooled weighted mean differences (WMD) with the REML model (**b**)


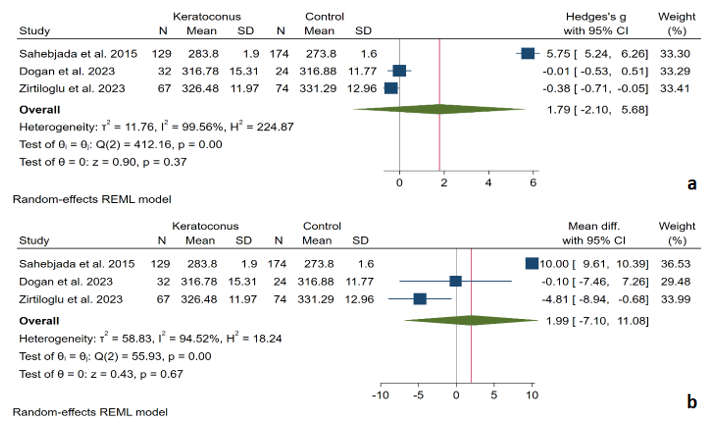


Supplementary Figure S23 - Forest plots of parafoveal macular thickness (overall) comparisons in eyes with KC and without KC; pooled Hedge's g with the REML model (**a**); pooled weighted mean differences (WMD) with the REML model (**b**)


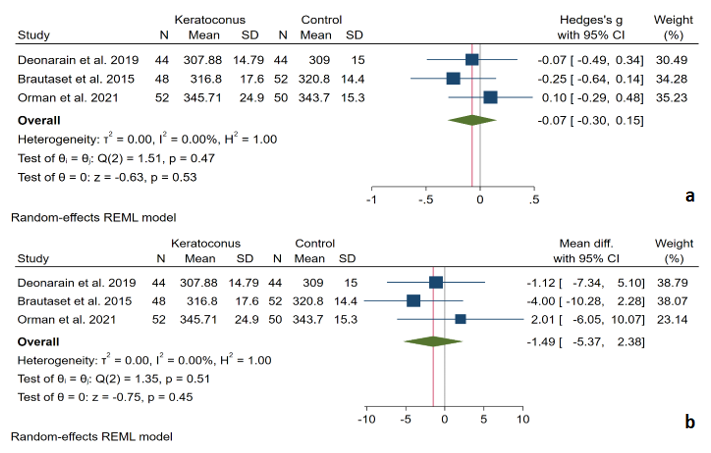
Supplementary Figure S24 - Forest plots of parafoveal macular thickness (inferior) comparisons in eyes with KC and without KC; pooled Hedge's g with the REML model (**a**); pooled weighted mean differences (WMD) with the REML model (**b**)


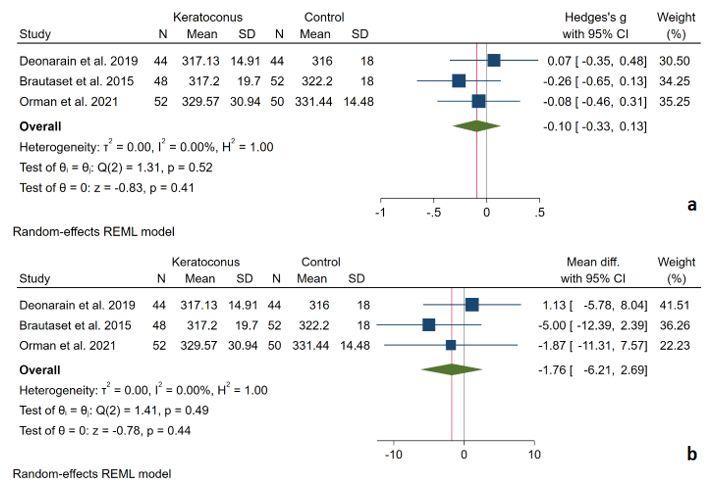
Supplementary Figure S25 - Forest plots of parafoveal macular thickness (nasal) comparisons in eyes with KC and without KC; pooled Hedge's g with the REML model (**a**); pooled weighted mean differences (WMD) with the REML model (**b**)


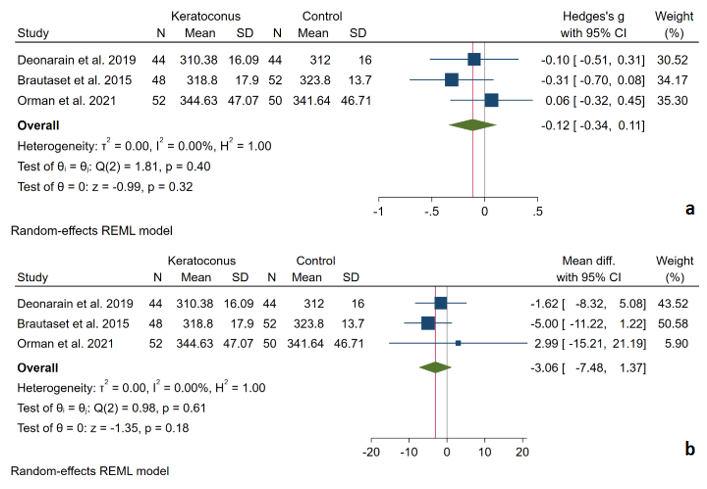
Supplementary Figure S26 - Forest plots of parafoveal macular thickness (superior) comparisons in eyes with KC and without KC; pooled Hedge's g with the REML model (**a**); pooled weighted mean differences (WMD) with the REML model (**b**)


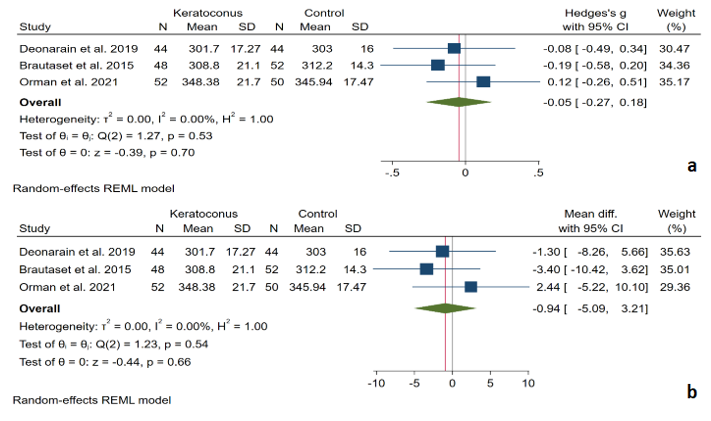
Supplementary Figure S27 - Forest plots of parafoveal macular thickness (temporal) comparisons in eyes with KC and without KC; pooled Hedge's g with the REML model (**a**); pooled weighted mean differences (WMD) with the REML model (**b**)


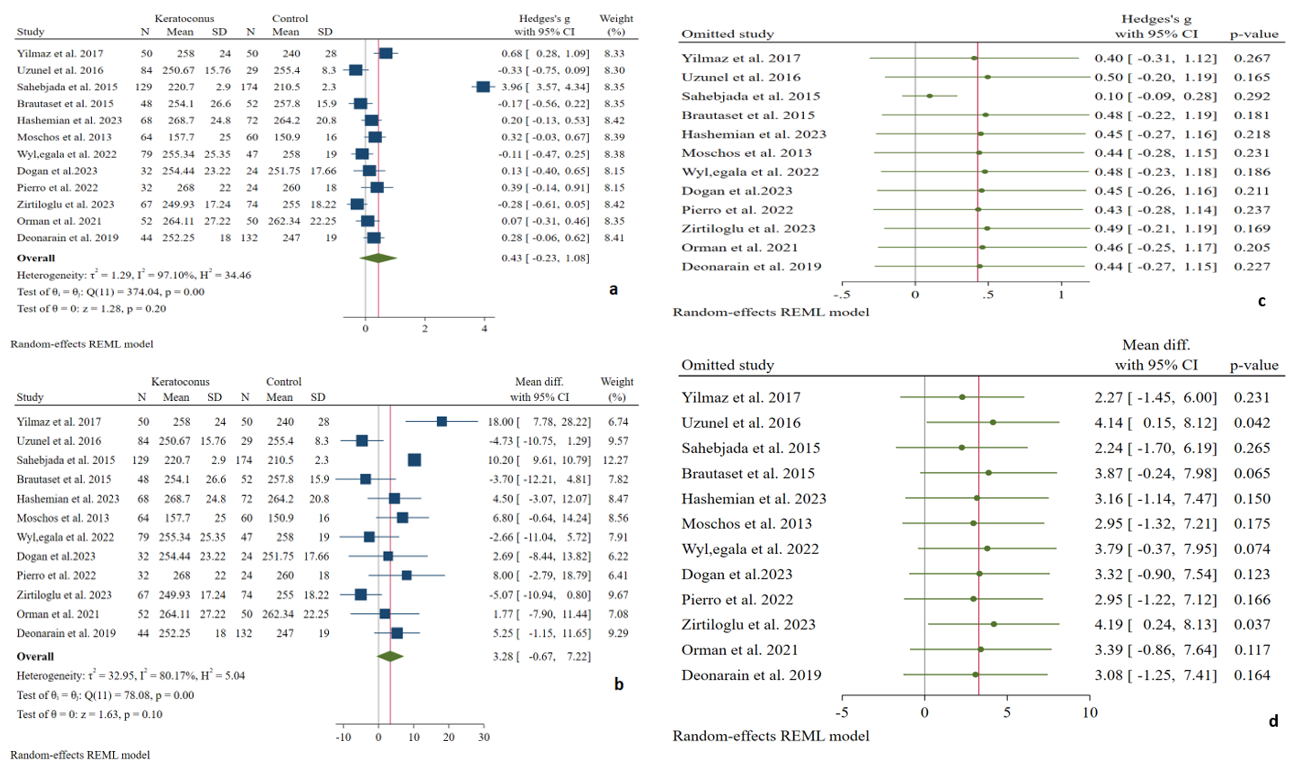
Supplementary Figure S28 - Forest plots of foveal macular thickness comparisons in eyes with KC and without KC; pooled Hedge's g with the REML model (**a**); pooled weighted mean differences (WMD) with the REML model (**b**) Leave-one-out sensitivity analysis on pooled Hedge's g (**c**) and weighted mean differences (WMD) (**d**) of foveal macular thickness


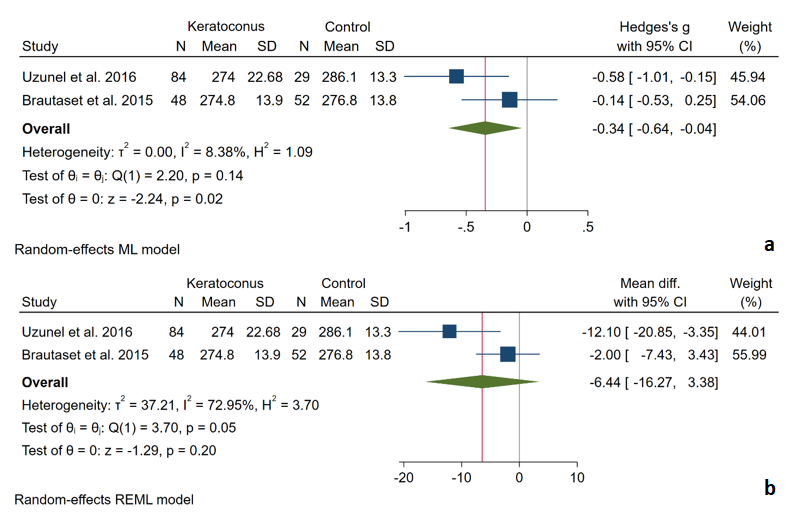
Supplementary Figure S29 - Forest plots of whole macular thickness comparisons in eyes with KC and without KC; pooled Hedge's g with the ML model (**a**); pooled weighted mean differences (WMD) with the REML model (**b**)


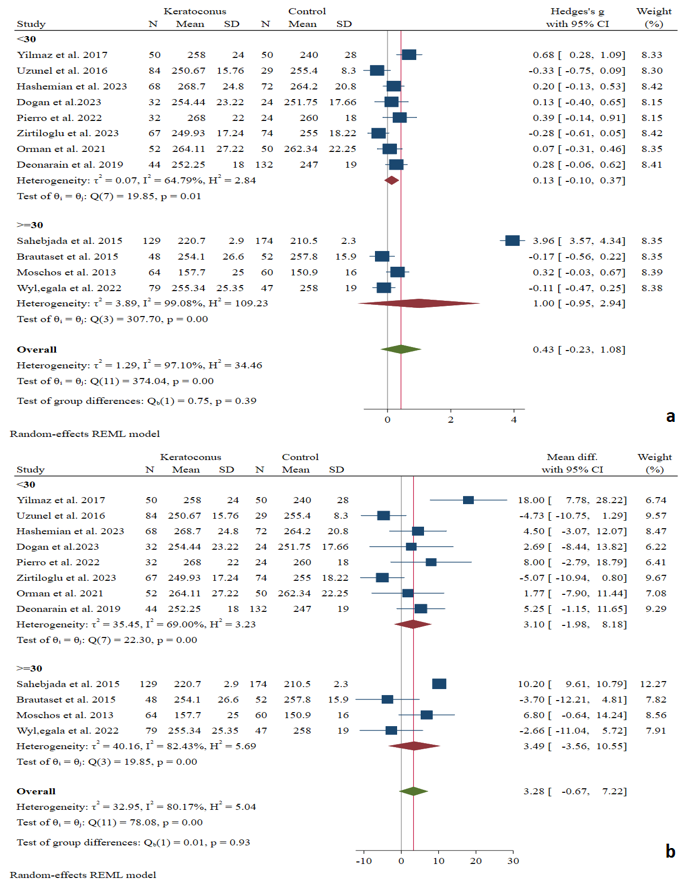
Supplementary Figure S30 - Forest plots of foveal macular thickness sub categories by age comparisons in eyes with KC and without KC; pooled Hedge's g with the REML model (**a**); pooled weighted mean differences (WMD) with the REML model (**b**)


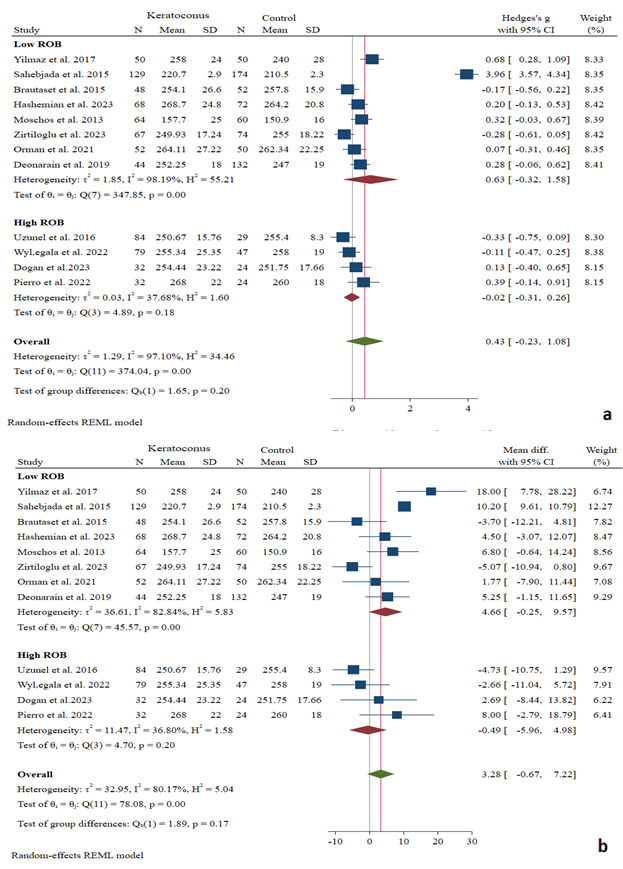


Supplementary Figure S31 - Forest plots of foveal macular thickness sub-categories by quality comparisons in eyes with KC and without KC; pooled Hedge's g with the REML model (**a**); pooled weighted mean differences (WMD) with the REML model (**b**)


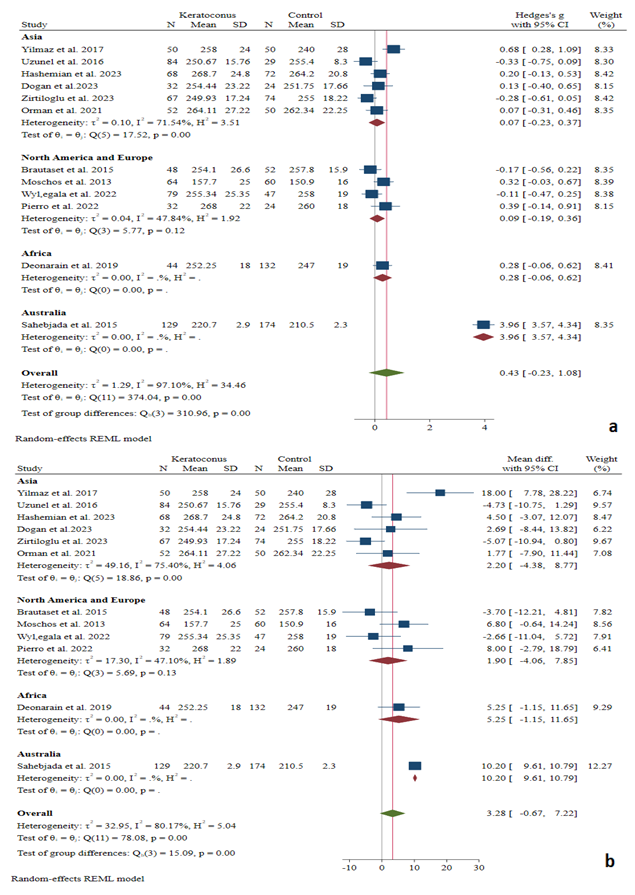


Supplementary Figure S32 - Forest plots of foveal macular thickness sub-categories by nation comparisons in eyes with KC and without KC; pooled Hedge's g with the REML model (**a**); pooled weighted mean differences (WMD) with the REML model (**b**)


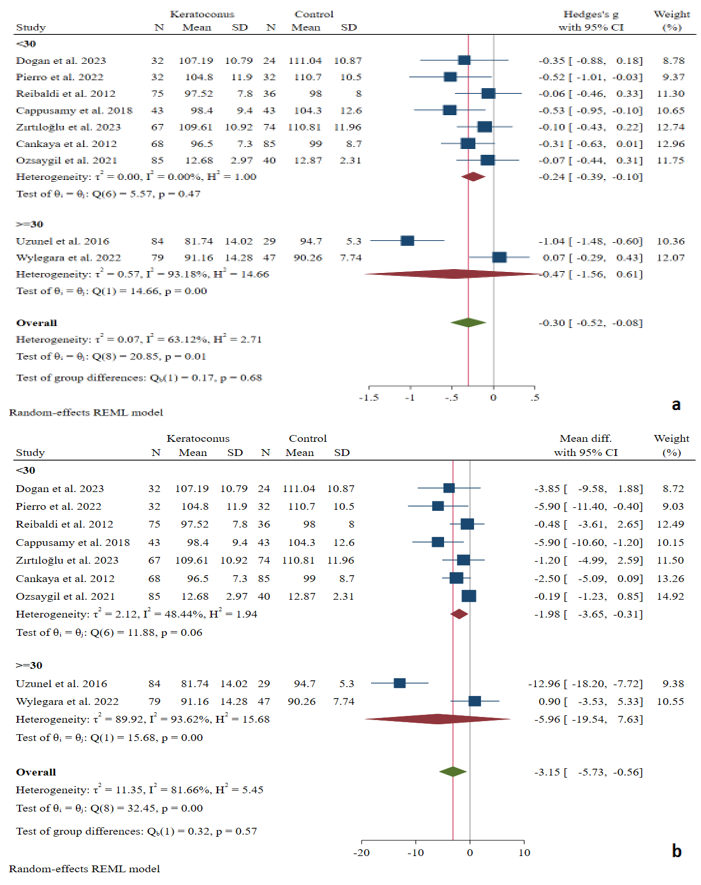


Supplementary Figure S33 - Forest plots of RNFL sub-categories by age comparisons in eyes with KC and without KC; pooled Hedge's g with the REML model (**a**); pooled weighted mean differences (WMD) with the REML model (**b**)


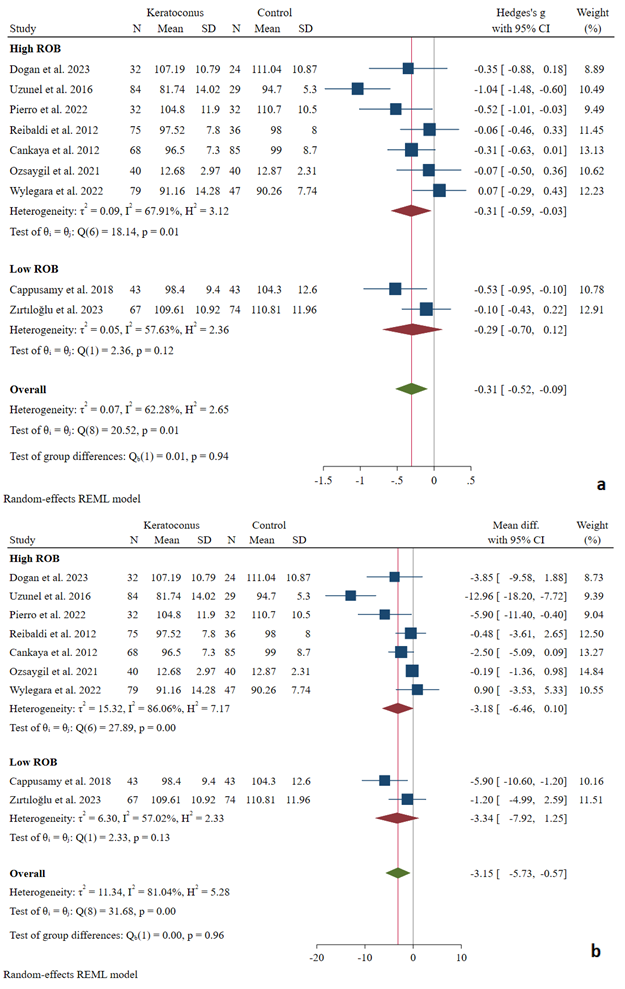
Supplementary Figure S34 - Forest plots of RNFL sub categories by quality comparisons in eyes with KC and without KC; pooled Hedge's g with the REML model (**a**); pooled weighted mean differences (WMD) with the REML model (**b**)


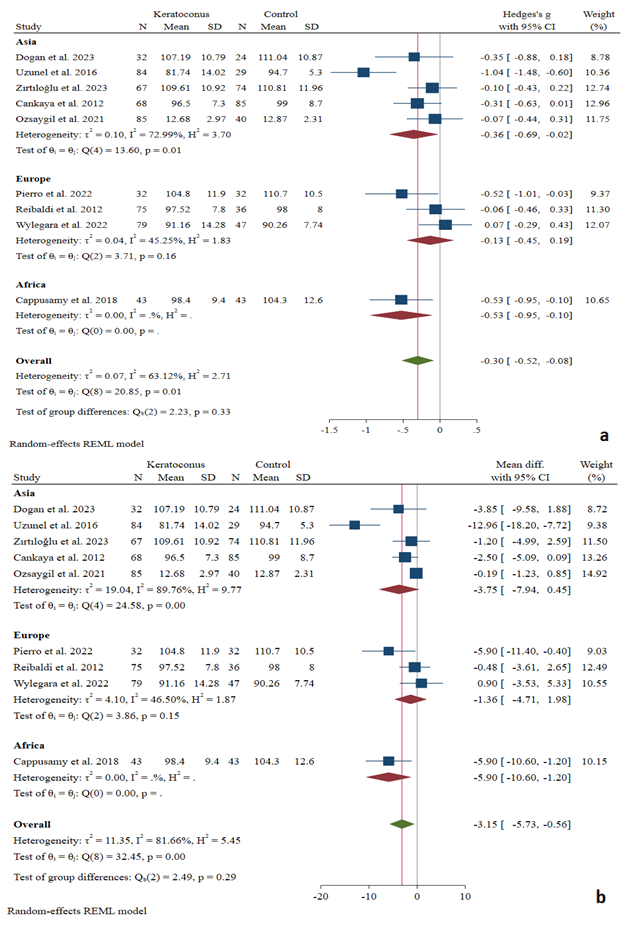


Supplementary Figure S35 - Forest plots of RNFL sub categories by nation comparisons in eyes with KC and without KC; pooled Hedge's g with the REML model (**a**); pooled weighted mean differences (WMD) with the REML model (**b)**
